# How electroacupuncture parameters shape brain responses

**DOI:** 10.3389/fnins.2026.1959062

**Published:** 2026-10-08

**Authors:** Ciqiu Tian, Qian Li, Fengxia Liang

**Affiliations:** 1 Hubei University of Chinese Medicine, Wuhan, Hubei, China; 2 Hubei Shizhen Laboratory, Wuhan, China; 3 Department of Integrated Chinese and Western Medicine, Liyuan Hospital Affiliated to Huazhong University of Science and Technology, Wuhan, Hubei, China

**Keywords:** current intensity, electroacupuncture, frequency, functional neuroimaging, neural circuits, neuroplasticity, stimulation parameters

## Abstract

Electroacupuncture (EA) is often treated as a uniform intervention, although its central effects vary with electrical parameters and stimulation site. This narrative review, supported by a structured search of PubMed/MEDLINE, Embase, Web of Science Core Collection, China National Knowledge Infrastructure, and SinoMed through August 4, 2026, synthesizes human and animal evidence on how frequency, current intensity, waveform, stimulation duration, and acupoint selection shape regional, network-level, electrophysiological, neurochemical, neurovascular, neuroimmune, and plasticity-related brain responses. Evidence was separated into direct comparative studies and an illustrative subset of single-protocol studies, with complementary transcutaneous electrical acupoint stimulation evidence considered separately. Frequency had the largest and most diverse evidence base. Low-, intermediate-, high-, and alternating-frequency protocols recruited overlapping sensorimotor, insular–cingulate, thalamic, limbic, hypothalamic, midbrain, and medullary systems, but differed in the relative distribution of regional activity and in associated neurochemical, neuronal, neurovascular, and plasticity-related processes. The available heterogeneous evidence does not establish a universally optimal frequency. The smaller current-intensity literature indicates recruitment thresholds, non-linear response ranges, and plateaus. Evidence for waveform and duration is limited and concentrated mainly in animal models of brain injury; waveform comparisons frequently involved concurrent differences in frequency composition and train structure, whereas duration effects varied by model and endpoint. Direct brain-level comparative evidence for pulse width remains insufficient. Acupoint and stimulation-site comparisons further showed site-, laterality-, and point-combination-related differences in cerebral, sensorimotor, neurovascular, and autonomic responses. Single-protocol studies characterized networks, receptors, cell populations, and defined circuits engaged under selected protocols, whereas parameter specificity was assessed from direct comparisons. Collectively, these findings support a multidimensional framework in which electrical dose and acupoint selection jointly shape brain responses. Future studies should use controlled, target-oriented comparisons, report complete stimulation characteristics, and link mechanistic brain measures to clinically relevant outcomes.

## 1 Introduction

Electroacupuncture (EA) has become widely used in experimental studies of acupuncture because the electrical stimulus delivered through inserted needles can be adjusted more consistently than manual needle manipulation. The stimulation protocol is defined by several electrical features, including frequency, current intensity, pulse width, waveform, and duration. Among these variables, frequency and current intensity have received the greatest experimental attention, particularly in studies of analgesia and central nervous system regulation (Zhao, 2008; Zhang et al., 2014). EA should therefore not be regarded as a uniform intervention, because different electrical settings may produce different neural effects even when the stimulated acupoints remain unchanged.

The cerebral effects of EA have been examined using fMRI, EEG, electrophysiological recording, neurochemical measurements, and markers of neuronal activation. These approaches have shown that EA can alter regional brain activity, neural oscillations, functional connectivity, and neuronal recruitment. Frequency has been the most directly investigated electrical parameter at the brain level. Comparative studies using low-, intermediate-, high-, or alternating-frequency stimulation have reported frequency-related differences in functional brain activity, immediate-early gene expression, neurochemical signaling, and electrophysiological responses (Zhang et al., 2003; Napadow et al., 2005; Qiu et al., 2015; Chai et al., 2025). Together, these studies indicate that stimulation frequency can alter measurable brain responses to EA.

Direct brain-level evidence for other electrical parameters is less extensive. Current intensity has been examined in a small number of functional imaging, cerebral perfusion, and neural circuit studies, whereas pulse width, waveform, and stimulation duration have seldom been tested as isolated variables (Shukla et al., 2011; Zhou et al., 2011; Liu et al., 2020). In many experiments, these features form part of a multicomponent stimulation protocol, making their individual contributions difficult to separate. The available literature therefore provides an uneven evidence base: frequency can be assessed across several experimental methods, while conclusions regarding intensity and other pulse characteristics rely on a smaller and more heterogeneous group of studies.

Previous reviews have mainly addressed the analgesic mechanisms of EA, including frequency-dependent neurochemical effects and peripheral, spinal, and supraspinal pathways (Zhou et al., 2023). Neuroimaging reviews have summarized EA-related changes in brain activity and functional connectivity but have not used electrical stimulation parameters as the principal framework for synthesis (Rodrigues et al., 2023). This review therefore examines how EA parameters and acupoint selection are related to regional and network-level brain responses, with frequency forming the main focus. Current intensity and other pulse characteristics are considered where direct brain-level evidence is available or where they affect the interpretation of frequency-related findings. By distinguishing direct comparative evidence from single-protocol evidence and presenting complementary TEAS evidence separately, this review further considers brain-response findings, standardized parameter reporting, and the design of future human electroacupuncture studies.

## 2 Search strategy and evidence classification

This narrative review was supported by a structured literature search. PubMed/MEDLINE, Embase, Web of Science Core Collection, China National Knowledge Infrastructure (CNKI), and SinoMed were searched from database inception to August 4, 2026. The primary searches combined terms relating to EA, electrical stimulation parameters, and brain-level outcomes. Studies addressing acupoint or stimulation-site selection were additionally identified through targeted supplementary searches and reference-list screening. Records were imported into NoteExpress (version 4.3.0.10710) and deduplicated. Titles and abstracts were screened independently by CT and FL, whereas full-text eligibility assessment and data extraction were performed independently by CT and QL; discrepancies were resolved through discussion. The complete database-specific search strategies and detailed screening and data-extraction procedures are provided in the Supplementary material, and the search sources and final exported record counts are summarized in Supplementary Table S1. The database searches identified 1,218 records, and the study-selection process is shown in Figure 1.

**Figure 1:**
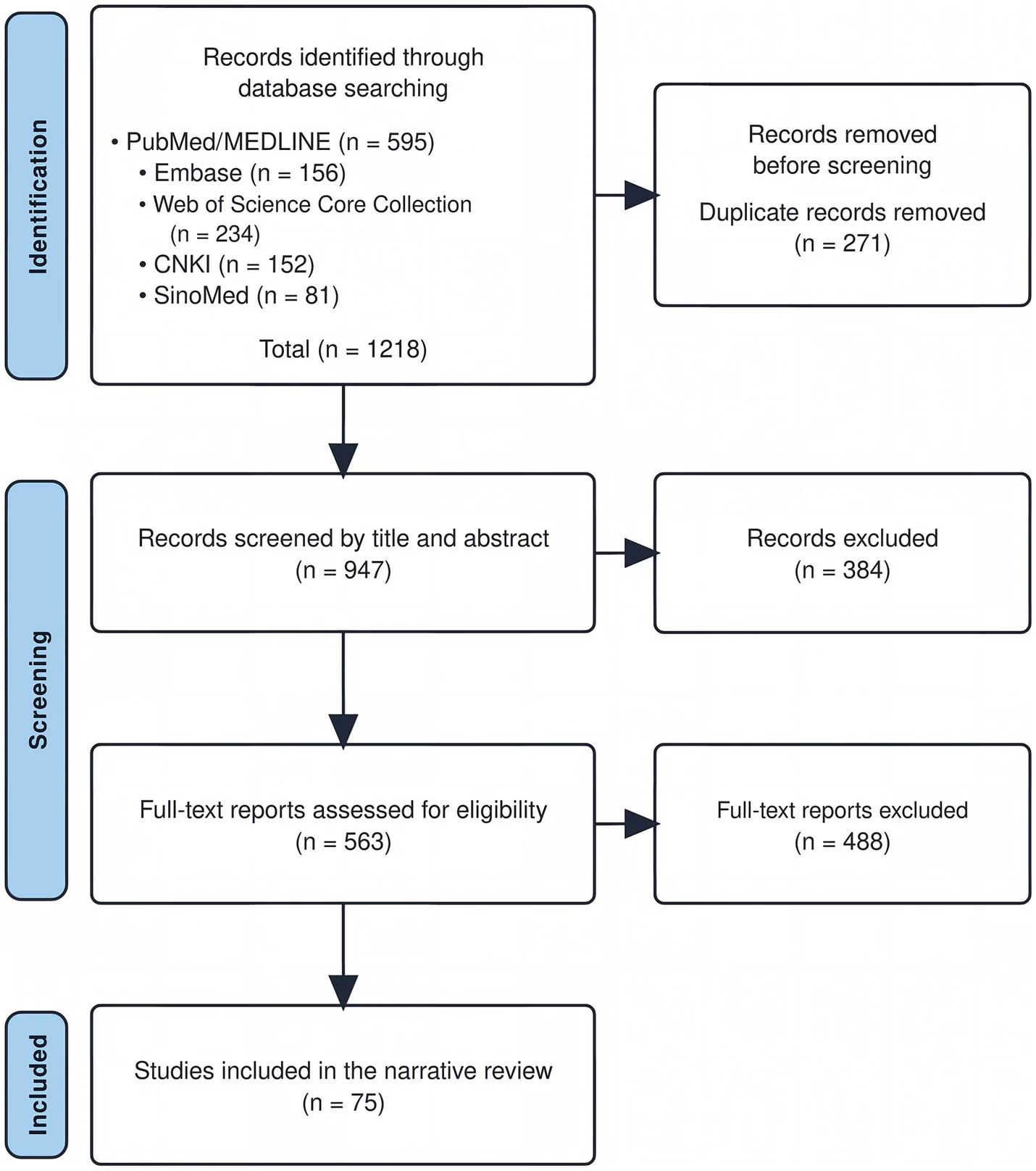
Flow diagram of the structured literature search and study selection. Searches of PubMed/MEDLINE, Embase, Web of Science Core Collection, China National Knowledge Infrastructure (CNKI), and SinoMed identified 1,218 records (PubMed/MEDLINE, *n* = 595; Embase, *n* = 156; Web of Science Core Collection, *n* = 234; CNKI, *n* = 152; SinoMed, *n* = 81). After removal of 271 duplicate records representing 168 duplicate clusters, 947 records underwent title-and-abstract screening; 384 records were excluded at this stage, and 563 full-text reports were assessed for eligibility. A further 488 reports were excluded after full-text assessment, leaving 75 sources retained in the narrative review. Of these, 69 contributed to the core brain-level evidence synthesis and six were used only for contextual interpretation.

Original human and animal studies were eligible when they reported a clearly defined EA protocol and at least one brain-level outcome. Studies confined to peripheral or spinal outcomes, studies reporting only clinical or behavioral outcomes without a corresponding brain-level measure, and reports with insufficient information on the electrical stimulation protocol were excluded from the core evidence synthesis. Needle-based EA was the primary intervention. TEAS studies were retained as complementary evidence only when they directly compared stimulation parameters and assessed cerebral responses, and were summarized separately. Detailed eligibility criteria are provided in Supplementary Table S2.

Evidence was classified at the study–outcome–comparison level. Direct comparative evidence required a within-study comparison of at least two levels or choices within one focal intervention dimension—frequency, current intensity, waveform, stimulation duration, or acupoint/site—for the same brain-level outcome. Comparisons in which another major stimulation feature also varied were coded as composite-protocol contrasts. Operational definitions and the corresponding co-varying characteristics are provided in Supplementary Tables S3, S4, respectively. “Direct” denotes the comparative study design rather than the statistical significance of the contrast.

Single-protocol evidence referred to brain-level findings obtained under one defined needle-based EA protocol without a within-study comparison of the focal intervention dimension for that outcome. It was used to identify associated brain regions, networks, circuits, receptors, cell populations, or cellular processes, but not to infer parameter or acupoint specificity or superiority. These studies formed an illustrative rather than exhaustive subset and were selected for brain-level informativeness and evidence coverage when they reported a clearly defined protocol, included a direct brain-level measurement or manipulation, and added anatomical or mechanistic information. Full coding rules and examples are provided in the Supplementary material. Individual study characteristics are presented in Tables 1, 2, and the relationships among intervention dimensions or defined protocols, assessment methods, brain systems, and response domains are summarized in Figure 2.

**Table 1:** Direct comparative evidence for needle-based electroacupuncture intervention dimensions and brain responses.

Study	Population or model	Brain assessment	Parameter comparison	Brain region(s) and parameter-dependent response
Frequency - human studies				
Napadow et al. (2005)	Human participants (*n* = 13; 100 Hz subset *n* = 8)	BOLD fMRI	2 vs. 100 Hz	2 and 100 Hz: S1/S2/aMCC ↑ amygdala/anterior hippocampus/cingulate/vmPFC ↓ 2 Hz > 100 Hz: regional recruitment 2 Hz: pontine raphe ↑
Ouyang et al. (2005)	Human - stroke (*n* = 47)	Cerebral perfusion imaging	2 vs. 2/15 vs. 100 Hz	Lesion perfusion: 2/15 Hz ≈ 100 Hz > 2 Hz
He et al. (2026)	Human - post-stroke hemiplegia (*n* = 134; groups *n* = 47, 42, and 45)	BOLD fMRI and FC	2 vs. 2/15 vs. 100 Hz	Lesion-side perfusion: 2/15 Hz > 2 and 100 Hz Contralesional primary motor cortex-cerebellum/insula FC: 2/15 Hz ↑
Frequency - animal studies				
Jang-Hern and Beitz (1993)	Sprague–Dawley rats (*n* = 22 total; frequency comparison *n* = 12, 6/group)	Fos mapping	4 vs. 100 Hz	4 and 100 Hz: multiple brainstem nuclei ↑ 4 Hz: cuneiform/dorsal-dlPAG/habenula/arcuate-lateral hypothalamus ↑ 100 Hz: rostrolateral ventral medulla ↑
Qiu et al. (2015)	Goats (*n* = 35; 7/group)	c-Fos/c-Jun IHC	2 vs. 60 vs. 100 Hz	Hypothalamic/descending nuclei: 60 Hz > 2 and 100 Hz NAc/lateral septum/caudate/basal amygdala/LC: NS
Guo et al. (2024)	Mice (*n* = 3–5/group)	Activity-dependent genetic labeling	2 vs. 100 Hz	2 Hz > 100 Hz: Cg1/S1/S2/insula/VPL/VL/PVT neuronal recruitment
Shen et al. (2025)	C57BL mice - trigeminal neuralgia with affective comorbidity (*n* = 8/group)	Whole-brain activity mapping	2 vs. 100 Hz	2 and 100 Hz: cortical–limbic-hypothalamic-midbrain-pontine activity altered 2 Hz > 100 Hz: broader PrL/BLA/PVN/VTA/vlPAG/LDT recruitment
Yao et al. (2020)	Mice - post-stroke dysphagia (*n* = 6–10/group, assay-dependent)	c-Fos IHC	2 vs. 50 vs. 100 Hz	Contralateral motor cortex c-Fos: 2 Hz > 50 and 100 Hz
Zhang et al. (2020)	Sprague–Dawley rats (*n* = 12/group; quantitative BBB assay *n* = 8/group)	BBB, perfusion, and c-Fos assays	Single-frequency vs. 2/100 Hz	2/100 Hz: BBB permeability ↑↑ Cortical perfusion/c-Fos ↑
Cheng et al. (2012)	Goats (*n* = 49; 7/group)	Opioid-peptide IHC	2 vs. 40 vs. 60 vs. 80 vs. 100 Hz	60 Hz: met-enkephalin/β-endorphin/dynorphin-A ↑↑ 40–100 Hz > 2 Hz: dynorphin-A
Guo et al. (1997)	Rats (*n* = 12; 3/group)	Regional mRNA assays	2 vs. 100 Hz	2 Hz: preproenkephalin mRNA ↑ (hypothalamic nuclei) 100 Hz: preprodynorphin mRNA ↑ (hypothalamic/parabrachial nuclei) POMC mRNA: NS
Qi et al. (2016)	Rats - acute visceral hyperalgesia (*n* = 8/group)	Opioid blockade	2 vs. 100 vs. 2/100 Hz	Naloxone blockade: 2 Hz complete 2/100 Hz partial 100 Hz minimal
Shen and Lai (2007)	Rats (sample size not reported)	Striatal microdialysis	3–30 Hz (3-Hz steps)	Striatal dopamine: 6/15 Hz ↑ during EA 21 Hz ↑ after EA Other tested frequencies: NS
Chang et al. (2004)	ICR mice (EA group *n* = 13)	Serotonin pharmacology	2 vs. 10 vs. 100 Hz	5-HT participation: 2/10/100 Hz 5-HT2 blockade: 100-Hz response ↑
Huang et al. (2019)	Rats - chronic constriction injury (*n* = 56 total; neural assays *n* = 6/group)	Neurochemical and receptor assays	2 vs. 15 vs. 50 Hz	15 Hz: hippocampal glutamate ↓; PAG GABA ↑ GABA receptors (hippocampus/PAG) ↑ Response > 2 and 50 Hz
Huo et al. (2023)	Mice - Alzheimer disease (*n* = 72; 12/group)	Hippocampal protein assays	2 vs. 15 vs. 30 Hz	Hippocampal IR/IRS-1/PI3K: 15/30 Hz > 2 Hz
Chen S. P. et al. (2016)	Rats - chronic constriction injury (*n* = 8/group for the frequency comparison; receptor assays *n* = 6/group)	Receptor assays and local blockade	2 vs. 2/15 vs. 100 Hz	M1 muscarinic/β2 nicotinic receptors: all ↑ α4 nicotinic mRNA: 2/15 Hz ↑ Local receptor blockade: EA response abolished
Xiang et al. (2026)	Mice - irritable bowel syndrome (*n* = 45; 9/group)	Hypothalamic CRF assays	2 vs. 15 vs. 2/15 Hz	Hypothalamic CRF/CRFR1 ↓ and CRFR2 ↑ (all) 2/15 Hz > 2 and 15 Hz: CRFR1 ↓, CRFR2 ↑
Zhang et al. (2023)	Sprague–Dawley rats - traumatic brain injury (*n* = 42; 6/group)	Oxidative, neuroimmune, and apoptosis assays	2 vs. 100 vs. 2/15 vs. 2/100 Hz	2/100 Hz: oxidative stress/pro-inflammatory signaling/NF-κB-COX-2/apoptosis ↓↓ Anti-inflammatory microglial markers ↑
Zhang et al. (2025)	Mice - methamphetamine neurotoxicity (*n* = 6–8/group)	BBB, histology, and microglial assays	2 vs. 10 Hz	CA1, 2 Hz vs. 10 Hz: BBB integrity ↑ Neuronal injury/microglial activation/pro-inflammatory signaling ↓
Zhang et al. (2009)	Rats (animal n not stated; 72 responsive units, 40 units tested)	Ventrobasal thalamic single-unit recording	2 vs. 100 Hz	Ventrobasal thalamic nociceptive firing: 2 and 100 Hz ↓ Receptive-field center: inhibition at 100 Hz > 2 Hz
Leite Ferreira et al. (2022)	Wistar rats - chronic neuropathic pain (*n* = 8/group)	Regional inactivation and stimulation	2 vs. 2/100 Hz	Infralimbic inactivation: responses to 2 and 2/100 Hz abolished Infralimbic stimulation: responses ↑
Wang et al. (1998)	Rats (*n* = 5/group)	Kappa-opioid receptor mRNA	Repeated 2 vs. 100 Hz	100 Hz: striatum/amygdala receptor mRNA ↓ 2 Hz: PAG/striatum ↓; amygdala ↑/biphasic
Li et al. (2016)	Rats - Alzheimer disease (*n* = 48; 8/group)	Hippocampal electrophysiology	2 vs. 50 Hz	Hippocampal LTP/LTD: 50 Hz > 2 Hz
Duan et al. (2018)	Rats - cerebral ischemia (*n* = 48; 16/group)	Neurotrophic-factor IHC	5 vs. 50 Hz	Ipsilesional CA3 BDNF/NGF-positive cells: 50 Hz > 5 Hz
Wang et al. (2016)	Rats - Alzheimer disease (*n* = 48; 8/group)	CA1 synaptic ultrastructure	2 vs. 30 vs. 50 Hz	CA1 synapse number/density: 50 Hz > 2 and 30 Hz
Xiao et al. (2013)	Rats - cerebral ischemia (*n* = 48 enrolled; *n* = 36 analyzed, 9/group)	Astrocyte and neuronal ultrastructure	15 vs. 30 vs. 100 Hz	Peri-infarct astrocyte preservation: 15/30 Hz > 100 Hz Neuronal ultrastructural damage: 15/30 Hz ↓
Chai et al. (2025)	Rats - propofol anesthesia (*n* = 36; 12/group)	EEG and neurochemical assays	† Composite: 2/15 vs. 50 Hz; manual-acupuncture comparator	2/15 Hz: delta-band activity altered; VLPO adenosine ↑ vs. 50 Hz 50 Hz: alpha/beta/theta activity altered
Fang et al. (2017)	Normal rats (*n* = 80 main cohort; 10/site-frequency subgroup)	NTS neuronal recording	† Composite: ST36 vs. ST25; 2 vs. 100 vs. 2/100 Hz	Point-frequency interaction in the NTS: ST36 2 Hz strongest excitation ST25 2 Hz: no significant excitation ST25 100 and 2/100 Hz: neuronal excitability/spike frequency ↓; strongest inhibition at 100 Hz
Current intensity - human studies				
Shukla et al. (2011)	Human - experimental heat pain (*n* = 10)	BOLD fMRI	Minimal vs. optimized intensity	Optimized > minimal: broader S2/dACC/premotor/amygdala/parietal/brainstem modulation Thalamic heat response ↓↓
Xiong et al. (2020)	Human - healthy participants (*n* = 12)	BOLD fMRI	0.65 ± 0.32 vs. 1.61 ± 0.38 mA	Low: left insula ↑; right precentral gyrus ↓ High: bilateral MTG/right insula/right IPL ↑; mPFC/sensorimotor/parietal/ITG ↓
Xiong et al. (2019)	Human participants (*n* = 22; 11/group)	Resting-state fMRI	0.50 ± 0.02 vs. 1.10 ± 0.10 mA	Post-EA amplitude - low: widespread ↓ High: widespread ↑
Huang et al. (2025)	Human - peripheral facial paralysis (*n* = 66 randomized; *n* = 61 completed)	Blink-reflex R2	Sensory-threshold vs. motion-threshold	R2 latency: motion threshold ↓↓ vs. sensory threshold ↓
Current intensity - animal studies				
Zhou et al. (2011)	Rats - middle cerebral artery occlusion (n ≥ 6 independent rats/parameter)	Regional CBF and infarct assessment	<0.6 to 1.2 mA	Ischemic CBF: <0.6 mA NS; 0.6–1.0 mA ↑; 1.2 mA plateau Infarct volume ↓ at effective range
Liu et al. (2020)	Mice (*n* = 5/group for neural assays; *n* = 20–33/group for survival)	Neural tracing, activation, and pathway manipulation	† Composite: low-intensity ST36 vs. high-intensity ST25	Low-intensity ST36: vagal-adrenal pathway ↑ High-intensity ST25: spinal-sympathetic pathway ↑
Yu et al. (2018)	Rats (*n* = 7/intensity)	SRD single-unit recording	0.5 to >6 mA	SRD: 0.5–1 mA NS 2–6 mA: spontaneous firing ↑ and nociceptive-firing inhibition ↑ >6 mA: plateau
Li et al. (2025)	Rats - inflammatory visceral hypersensitivity (*n* = 8/group for neuronal recordings; *n* = 6/group for behavior)	Medullary neuronal recording	0.5 vs. 1 vs. 2 vs. 3 vs. 6 mA	SRD: 0.5/1 mA weak 2 < 3 < 6 mA: nociceptive-firing inhibition/responsive neurons ↑
Gong et al. (2022)	Rats (*n* = 16/group for intensity comparison; calcium imaging *n* = 4/group)	BBB, c-Fos, and calcium imaging	1 vs. 3 vs. 5 mA	BBB permeability/frontal c-Fos: 3 mA > 1 and 5 mA S1 glutamatergic Ca2 + events: 3/5 mA > 1 mA 3–5 mA plateau
Liu et al. (2021)	Mice (*n* = 3–5/group for neural assays; n ≥ 20/group for survival)	Neural tracing, cholinergic activation, and afferent ablation	† Composite: 0.5-mA ST36 vs. 3.0-mA ST36/ST25	0.5-mA ST36: PROKR2-positive afferents → DMV/vagal-adrenal pathway ↑ 3.0-mA ST36 or ST25: spinal sympathetic pathway ↑
Waveform				
Chen Y. et al. (2016)	Rats - sepsis-associated brain injury (*n* = 20/group for survival analysis)	Histology and molecular assays	† Composite: continuous 2 Hz vs. intermittent 2/0 Hz vs. dilatational 2/15 Hz	2/15-Hz dilatational > intermittent > continuous: cerebral edema/BBB leakage/oxidative stress/inflammation/apoptosis ↓ Hippocampal neuronal preservation ↑
Yu et al. (2026)	Rats - cerebral ischemia–reperfusion (*n* = 70 selected; *n* = 48 successfully modeled, 12/group)	Infarct and molecular assays	† Composite: continuous 15 Hz vs. discontinuous 15-Hz trains vs. dense-disperse 3/15 Hz	All: infarct volume/NLRP3-pyroptosis ↓ Dense-disperse > continuous; selected caspase-1/ASC markers: dense-disperse > discontinuous
Stimulation duration				
Zhang et al. (2018)	Rats - recovery after middle cerebral artery occlusion (*n* = 16/group)	BBB and water-content assays	Multiple short durations; 8 min maximal	BBB permeability: 8 min maximal ↑ NGF brain entry ↑ Brain water content: NS
Zhou et al. (2013b)	Rats - acute cerebral ischemia (*n* = 16–45/group)	CBF, infarct, and opioid blockade	5 vs. 15 vs. 30 vs. 45 min	Infarct volume: ↓ to 30 min; effect lost at 45 min Reperfusion: 5–30 min ↑; 45 min ↓ δ-opioid blockade: 30-min effect abolished
Zhang et al. (2007)	Rats - cerebral ischemia–reperfusion (*n* = 40; 8/group)	Glutamate and aspartate assays	10 vs. 30 vs. 60 min	Glutamate: 10/30/60 min ↓ Aspartate: 60 min ↓
Chuang et al. (2013)	Rats - traumatic brain injury (*n* = 132 total; 6/group per outcome)	rCBF, lesion, and apoptosis assays	30 vs. 60 min	60 min > 30 min: cortical lesion volume/apoptosis/TGIF ↓ rCBF ↑
Acupoint or stimulation-site comparisons				
Liu et al. (2011)	Healthy volunteers (*n* = 28)	Whole-brain power analysis	GB37 vs. KI8	Whole-brain power: different patterns in PAG, occipital cortex, and temporal cortex Sustained response: differences in occipital cortex, dorsolateral/medial prefrontal cortices, ACC, and insula
Maeda et al. (2013)	Human - carpal tunnel syndrome (*n* = 59; groups *n* = 22, 18, and 19)	Event-related BOLD fMRI	Local PC7/TE5 vs. distal contralateral SP6/LR4 vs. sham	Local: insula/S2 activation ↑; ipsilateral S1/inferior temporal gyrus deactivation ↑ Distal: bilateral S2 activation ↑; PCC deactivation ↑
Maeda et al. (2017)	Human - carpal tunnel syndrome (*n* = 80 randomized; healthy controls *n* = 34)	Longitudinal BOLD/DTI	Local vs. distal stimulation	Fractional anisotropy increased adjacent to the hand vs. leg representations of the ipsilesional S1 after local vs. distal treatment, respectively
Fu et al. (2023)	Human - swallowing task (*n* = 27; 9/group)	BOLD fMRI, cortical mapping, and FC	† Composite: HT5 + GB20 vs. HT5 alone vs. GB20 alone	Combination: left M1 activation and interhemispheric motor connectivity ↑ vs. HT5 alone Sensorimotor-premotor connectivity ↑ vs. GB20 alone
Fang et al. (2017)	Normal rats (*n* = 80 main cohort; 10/site-frequency subgroup)	NTS neuronal recording	† Composite: ST36 vs. ST25 across 2, 100, and 2/100 Hz	ST36: 2-Hz excitation strongest ST25: 100- and 2/100-Hz inhibition; 100 Hz strongest
Liu et al. (2020)	Mice (*n* = 5/group for neural assays; *n* = 20–33/group for survival)	Neural tracing, activation, and pathway manipulation	† Composite: ST36 low intensity vs. ST25 high intensity	ST36 low intensity: vagal-adrenal pathway ↑ ST25 high intensity: spinal-sympathetic pathway ↑
Liu et al. (2021)	Mice (*n* = 3–5/group for neural assays; n ≥ 20/group for survival)	Neural tracing, cholinergic activation, and afferent ablation	† Composite: ST36 0.5 mA vs. ST36/ST25 3 mA at 10 Hz	ST36: PROKR2-positive deep-fascia afferents and DMV/vagal-adrenal recruitment at 0.5 mA ST36/ST25: ChAT-positive spinal sympathetic recruitment at 3 mA
Lee et al. (2020)	Sprague–Dawley rats (*n* = 18; 6/group)	Metabolomic analysis	Matched 2-Hz ST36 vs. PC6	ST36: cortical/hippocampal adrenaline ↑; hippocampal arginine ↑ PC6: cortical/hippocampal GABA and glycine ↓; hippocampal glutamate ↓
Wang et al. (2016)	Rats - cerebral ischemia–reperfusion (*n* = 90; 18/group; assay subsets *n* = 6–12/group)	BKCa-channel and histology assays	GV26 vs. nearby non-acupoint; 2 Hz	GV26: cortical BKCa expression and pathological injury ↓ Nearby non-acupoint: no corresponding change
Zhou et al. (2013a)	Rats - right middle cerebral artery occlusion (*n* = 24–60/group)	CBF and infarct assessment	Matched 5/20-Hz, 1.0-mA protocols at different point sets	GV20 + GV26 or left, lesion-contralateral LI11 + PC6: ischemic cortical CBF ↑ and infarct volume ↓ Right LI11 + PC6 or left GB34 + SP6: CBF/infarct NS
Deng et al. (2016)	Healthy participants (*n* = 33)	Regional homogeneity fMRI	GV20 vs. sham point	ReHo ↑: orbitofrontal, middle cingulate, precentral cortex, precuneus ReHo ↓: ACC, SMA, thalamus, putamen, cerebellum

↑, increase/activation/strengthening; ↓, decrease/deactivation/reduction; >, greater response; NS, no statistically significant change or difference as reported by the original study. Arrows and comparison symbols summarize the reported direction only and do not indicate effect size, evidence strength, or causal certainty. †, composite contrast in which another protocol feature (stimulation site, number of stimulated points, pulse train, waveform, or comparator intervention) also differed; complete co-varying protocol fields are reported in the Supplementary material. A study may appear in more than one focal-dimension section when separate outcomes were coded. The table contains 53 unique reports represented by 56 coded rows. These counts exclude complementary TEAS studies. 5-HT, 5-hydroxytryptamine; ACC, anterior cingulate cortex; aMCC, anterior midcingulate cortex; BBB, blood–brain barrier; BDNF, brain-derived neurotrophic factor; CBF, cerebral blood flow; ChAT, choline acetyltransferase; CRF/CRFR, corticotropin-releasing factor/receptor; dACC, dorsal anterior cingulate cortex; DMN, default mode network; EEG, electroencephalography; FC, functional connectivity; fMRI, functional magnetic resonance imaging; GABA, gamma-aminobutyric acid; IHC, immunohistochemistry; IPL, inferior parietal lobule; LFP, local-field potential; LTP/LTD, long-term potentiation/depression; MCAO, middle cerebral artery occlusion; NAc, nucleus accumbens; NGF, nerve growth factor; NPY, neuropeptide Y; NTS, nucleus of the solitary tract; PAG, periaqueductal gray; PCC, posterior cingulate cortex; PROKR2, prokineticin receptor 2; ReHo, regional homogeneity; rCBF, regional cerebral blood flow; S1/S2, primary/secondary somatosensory cortex; SMA, supplementary motor area; SRD, subnucleus reticularis dorsalis; TGIF, transforming growth interacting factor; vlPAG, ventrolateral periaqueductal gray; vmPFC, ventromedial prefrontal cortex.

**Table 2:** Illustrative single-protocol evidence from needle-based electroacupuncture.

Study	Population or model	Defined EA protocol	Brain assessment/manipulation	Brain region or circuit	Main brain-level finding
Fisher et al. (2021)	Human - carpal tunnel syndrome (CTS *n* = 64 at baseline and *n* = 47 post-treatment; healthy controls *n* = 28)	Repeated 2-Hz EA	Resting-state FC	S1-pulvinar-anterior hippocampal network	S1-pulvinar/anterior hippocampus FC altered; normalization associated with later clinical improvement
Wang et al. (2024)	Human - first-episode major depressive disorder (*n* = 28; 14/group)	1-Hz EA	Resting-state fMRI	Temporal-frontal-hippocampal and cerebellar-occipital networks	Left MTG/posterior cerebellum ALFF ↑ MTG-frontal/hippocampal FC ↑ Cerebellum-calcarine FC ↓
Boyang et al. (2024)	Human - stroke-related sleep disorder (*n* = 72 trial participants; fMRI analysis *n* = 16 patients and *n* = 15 controls)	2-Hz scalp EA	Resting-state fMRI	Frontal/default-mode and parietal-occipital regions	Medial SFG/precuneus/PCC ↓ Angular gyrus/IPL/middle occipital gyrus ↑
Zha et al. (2024)	Human - subjective tinnitus (tinnitus *n* = 34; controls *n* = 34)	2/50-Hz EA	Insula-seed FC	Insula-centered network	Insula-frontal/paracentral FC ↓
Zhang et al. (2024)	Human - subjective tinnitus (tinnitus *n* = 40; controls *n* = 40)	2/50-Hz acupuncture protocol	ACC-seed fMRI FC	ACC-centered network	ACC-frontal/temporal/thalamic FC altered
Bao et al. (2017)	Human - Crohn disease (*n* = 65 randomized; fMRI analysis *n* = 18 EA and *n* = 20 moxibustion)	Repeated 2/100-Hz EA	Resting-state fMRI FC	Hippocampus-aMCC-insula network	Hippocampus-aMCC/insula FC ↑
Xu et al. (2024)	5xFAD mice (*n* = 16 total)	2-Hz EA	Functional-connectivity imaging	Hippocampal-limbic-neocortical network	Hippocampal FC and limbic-neocortical FC ↑
Cai et al. (2025)	Rats - remifentanil-induced hyperalgesia (*n* = 5/group)	2-Hz EA	Single-unit and LFP recording	VPL/S1/insula/ACC network	Neuronal hyperactivity ↓ Theta-band LFP ↓
Liang et al. (2010)	Rats - morphine conditioned-place preference (*n* = 218 total across three subexperiments)	Repeated 2-Hz EA	NAc neurochemistry and central receptor blockade	Nucleus accumbens/central opioid system	NAc enkephalin/preproenkephalin mRNA ↑ μ/δ blockade: response abolished κ blockade: NS
Wang et al. (2011)	Morphine-dependent rats (*n* = 4–5/group)	Repeated 100-Hz EA	Regional mRNA and p-CREB assays	PAG/hypothalamus	Preprodynorphin mRNA restored ↑ p-CREB ↓
Wei et al. (2021)	Mice - neuropathic pain (*n* = 4–6/group, assay-dependent)	2-Hz EA	Cell-type-specific cortical recording and manipulation	Primary somatosensory cortex microcircuit	Pyramidal/VIP-positive neurons ↓ Somatostatin-positive interneurons ↑
Ma et al. (2025)	Mice - chronic unpredictable stress (*n* = 48; 12/group)	2-Hz EA	Pyramidal-neuron E/I assessment	dlPFC/mPFC/ventral CA1	E/I ratio ↓
Zhang et al. (2026)	Sprague–Dawley rats - neuropathic pain (*n* = 8/group)	2-Hz EA	Fiber-optic calcium recording and chemogenetics	rACC-vlPAG pathway	rACC CaMKII-positive activity↑ rACC-vlPAG activation: EA response reversed
Liu et al. (2021)	Mice (*n* = 3–5/group for neural assays; n ≥ 20/group for survival)	Selected 0.5-mA ST36 EA	Neural tracing, cholinergic activation, and afferent ablation	DMV/vagal-adrenal pathway	DMV cholinergic neurons ↑ PROKR2-positive afferent ablation: DMV/adrenal responses abolished
Zhang et al. (2020)	Sprague–Dawley rats (*n* = 4–6/group)	Selected composite 2/100-Hz EA	Cortical perfusion and c-Fos assays	Cortex/BBB	Follow-up under the selected 2/100-Hz protocol: cortical perfusion ↑ and cortical c-Fos expression ↑
Zhang et al. (2023)	Sprague–Dawley rats - traumatic brain injury (*n* = 48; 12/group)	Selected composite 2/100-Hz EA	Oxidative, neuroimmune, and apoptosis assays	Cortex/microglia	Follow-up after protocol screening: oxidative stress and NF-κB/COX-2 signaling ↓ Microglial markers shifted toward an anti-inflammatory profile Neuronal apoptosis ↓

↑, increase/activation/strengthening; ↓, decrease/deactivation/reduction; >, greater response; NS, no statistically significant change or difference as reported by the original study. Arrows and comparison symbols summarize the reported direction only and do not indicate effect size, evidence strength, or causal certainty. The 16 single-protocol studies are illustrative rather than exhaustive and do not establish parameter or acupoint specificity or superiority. Complementary TEAS studies are summarized separately and are not included. ACC, anterior cingulate cortex; ALFF, amplitude of low-frequency fluctuations; aMCC, anterior midcingulate cortex; BBB, blood–brain barrier; BOLD, blood-oxygen-level-dependent; CaMKII, calcium/calmodulin-dependent protein kinase II; DMV, dorsal motor nucleus of the vagus; E/I, excitatory/inhibitory; FC, functional connectivity; fMRI, functional magnetic resonance imaging; LFP, local-field potential; MTG, middle temporal gyrus; NAc, nucleus accumbens; NF-κB, nuclear factor kappa B; PAG, periaqueductal gray; PCC, posterior cingulate cortex; PROKR2, prokineticin receptor 2; rACC, rostral anterior cingulate cortex; S1/S2, primary/secondary somatosensory cortex; vlPAG, ventrolateral periaqueductal gray; VPL, ventral posterolateral thalamic nucleus; VIP, vasoactive intestinal peptide.

**Figure 2:**
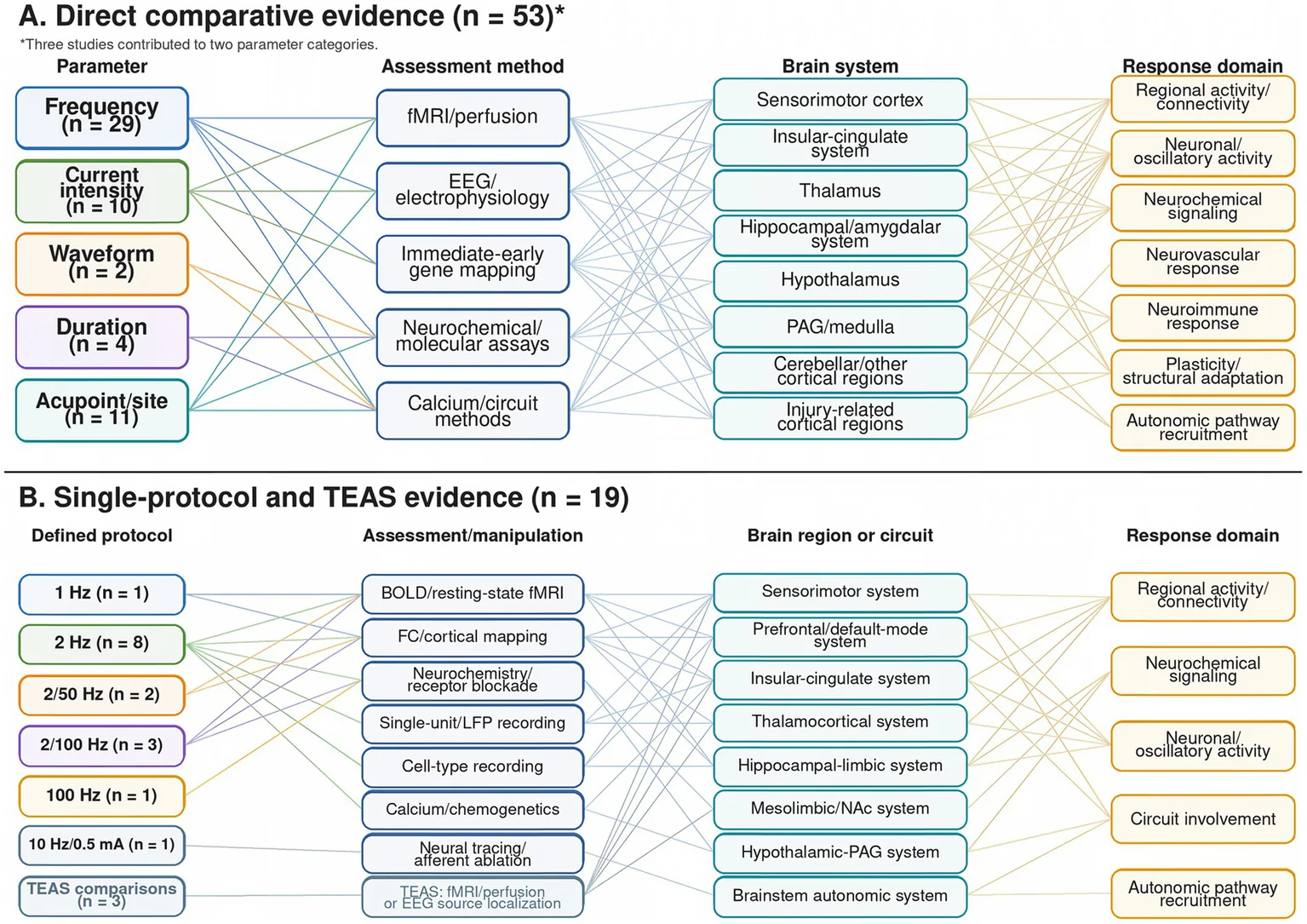
Evidence map of brain-level studies grouped by evidence type and focal intervention dimension. Mapping of electroacupuncture parameters and protocols to neural assessment methods, brain systems, and response domains. **(A)** Direct comparative evidence from 53 unique needle-based electroacupuncture studies. Parameter-category counts sum to 56 because three studies were each classified into two parameter categories. **(B)** Evidence from 16 single-protocol needle-based electroacupuncture studies and three transcutaneous electrical acupoint stimulation comparisons (*n* = 19). Connecting lines indicate reported links between adjacent domains. EA, electroacupuncture; TEAS, transcutaneous electrical acupoint stimulation; BOLD, blood oxygen level-dependent; fMRI, functional magnetic resonance imaging; EEG, electroencephalography; FC, functional connectivity; LFP, local field potential; PAG, periaqueductal gray; NAc, nucleus accumbens.

## 3 Frequency

### 3.1 Frequency patterns

Low-frequency EA has most commonly been delivered at 2 or 4 Hz, whereas 100 Hz is the most frequently used high-frequency condition. Intermediate-frequency studies have used values including 10, 15, 30, 40, 50, 60, and 80 Hz, either as individual conditions or within multi-level frequency designs. Alternating-frequency protocols deliver two frequencies successively within the same stimulation session. The most frequently examined combinations are 2/15, 2/50, and 2/100 Hz. Accordingly, these alternating-frequency conditions are described below as composite frequency protocols.

### 3.2 Direct comparative evidence

#### 3.2.1 Regional brain responses

##### 3.2.1.1 Human evidence

Direct neuroimaging comparisons have identified substantial overlap between low- and high-frequency cerebral responses, together with frequency-related recruitment of additional regions. During needle-based EA at ST36, both 2- and 100-Hz stimulation increased signals in the leg representation of the primary somatosensory cortex, secondary somatosensory cortex, and anterior midcingulate cortex and decreased signals in the amygdala, anterior hippocampus, subgenual and retrosplenial cingulate cortices, and ventromedial prefrontal cortex. In the group-level analysis, brain regions showing significant signal increases at 2 Hz but not at 100 Hz included the ipsilateral orbitofrontal cortex, ipsilateral anterior and posterior midcingulate cortices, contralateral anterior insula, ipsilateral posterior insula, contralateral anterior putamen, ipsilateral anterior thalamus, and the pontine raphe area (Napadow et al., 2005).

Clinical comparisons in patients with stroke have primarily identified frequency-related differences in cerebral perfusion and motor-network connectivity. Scalp EA at 2, alternating 2/15, or 100 Hz increased cerebral perfusion within the lesion region, with larger changes after the composite 2/15-Hz and 100-Hz protocols than after 2 Hz (Ouyang et al., 2005). In a subsequent study of 134 patients with post-stroke hemiplegia, the lesion-side regional-to-whole-brain perfusion ratio increased after all three protocols but was higher after the composite 2/15-Hz protocol than after either single-frequency protocol. The 2/15-Hz group also showed greater connectivity changes between the contralesional primary motor cortex and the left cerebellar lobules VI and VIII and right insula (He et al., 2026).

##### 3.2.1.2 Animal evidence

At the brainstem level, both 4- and 100-Hz EA increased Fos expression in the lateral parabrachial nucleus, substantia nigra, nucleus raphe pallidus, dorsal raphe nucleus, locus coeruleus, posterior pretectal nucleus, and ventrolateral periaqueductal gray. Four-hertz stimulation additionally recruited the cuneiform nucleus, dorsal and dorsolateral periaqueductal gray, habenular nucleus, arcuate hypothalamic nucleus, and lateral hypothalamic regions, whereas the rostrolateral ventral medulla was the principal brainstem region activated by 100 Hz but not by 4 Hz (Jang-Hern and Beitz, 1993). A wider frequency comparison identified a different regional profile. In goats receiving 2-, 60-, or 100-Hz EA, 60-Hz stimulation induced greater c-Fos or c-Jun expression in most of the examined regions, including the supraoptic, paraventricular, arcuate, and ventromedial hypothalamic nuclei, periaqueductal gray, parabrachial nucleus, nucleus raphe magnus, gigantocellular reticular nucleus, and nucleus tractus solitarius. No significant frequency-related differences were detected in the nucleus accumbens, lateral septal area, caudate nucleus, basal amygdala, or locus coeruleus (Qiu et al., 2015). Cortical and thalamic recruitment has been examined using activity-dependent genetic labeling. Comparison of 2- and 100-Hz EA at ST36 showed greater neuronal recruitment with 2 Hz in cingulate cortex area 1, the primary and secondary somatosensory cortices, and the insular cortex. Within the thalamus, 2-Hz stimulation recruited more neurons in the ventral posterolateral and ventrolateral nuclei and increased labeling in the paraventricular nucleus relative to sham stimulation (Guo et al., 2024). In a model of trigeminal neuralgia with anxiety- and depression-like behaviors, both 2- and 100-Hz EA altered neuronal activity across cortical, limbic, hypothalamic, midbrain, and pontine regions. The 2-Hz protocol recruited a broader set of regions, including the prelimbic cortex, basolateral amygdala, paraventricular hypothalamic nucleus, ventral tegmental area, ventrolateral periaqueductal gray, and laterodorsal tegmental nucleus (Shen et al., 2025). A more focal motor-cortical response was observed after post-stroke dysphagia, in which comparison of 2-, 50-, and 100-Hz EA showed increased c-Fos-positive neurons in the contralateral motor cortex under the 2-Hz condition (Yao et al., 2020). Frequency also modified neurovascular responses. Comparison of single-frequency protocols with the composite 2/100-Hz protocol showed that 2/100-Hz EA produced the largest transient increase in blood–brain barrier permeability, which returned toward baseline after stimulation ended (Zhang et al., 2020).

#### 3.2.2 Frequency-related neural processes

##### 3.2.2.1 Neurochemical signaling

Frequency-dependent opioid effects were observed at peptide, transcriptional, and pharmacological levels. Across 2–100 Hz, 60-Hz EA produced the broadest regional increases in met-enkephalin, β-endorphin, and dynorphin-A immunoreactivity, while dynorphin-A responses were more prominent at 40–100 than at 2 Hz (Cheng et al., 2012). At the transcriptional level, 2 Hz preferentially increased preproenkephalin mRNA in several hypothalamic nuclei and the nucleus of the lateral lemniscus, whereas 100 Hz increased preprodynorphin mRNA in hypothalamic and parabrachial regions (Guo et al., 1997). Pharmacological blockade further distinguished opioid involvement across frequency protocols. Systemic naloxone abolished the suppression of colorectal-distension responses produced by 2-Hz EA, partially attenuated the composite 2/100-Hz response, and had little effect on the 100-Hz condition (Qi et al., 2016).

Non-opioid neurotransmitter systems display different frequency profiles. Striatal microdialysis across frequencies from 3 to 30 Hz showed immediate increases in extracellular dopamine at 6 and 15 Hz, whereas the response to 21 Hz emerged after stimulation had ended. Frequencies of 3, 9, 12, 18, 24, 27, and 30 Hz did not produce comparable changes under the same conditions (Shen and Lai, 2007). Central serotonergic signaling contributed to analgesia at 2, 10, and 100 Hz. Inhibition of serotonin synthesis attenuated the response to all three frequencies, and antagonists of 5-HT₁A and 5-HT₃ receptors similarly reduced each response. In contrast, 5-HT₂ receptor blockade potentiated the effect of 100-Hz EA. This pattern indicates shared serotonergic participation across frequencies, together with a distinct interaction between 5-HT₂ signaling and high-frequency stimulation (Chang et al., 2004). Excitatory and inhibitory amino-acid responses were most prominent at an intermediate frequency in a chronic constriction injury model. Under the 15-Hz protocol, EA decreased hippocampal glutamate and increased γ-aminobutyric acid in the periaqueductal gray, while comparison of the 2-, 15-, and 50-Hz groups showed higher GABA-receptor expression in both regions at 15 Hz. The examined N-methyl-D-aspartate receptor subunits did not show a corresponding response (Huang et al., 2019).

Frequency-related effects also extended to hippocampal insulin signaling. In an Alzheimer’s disease mouse model, 2-, 15-, and 30-Hz EA were compared under otherwise matched treatment conditions. Fifteen- and 30-Hz stimulation increased hippocampal insulin receptor, insulin receptor substrate-1, and phosphatidylinositol 3-kinase protein expression relative to both untreated model animals and the 2-Hz group (Huo et al., 2023). Cholinergic receptor signaling showed an additional frequency-related pattern in the hippocampus. In rats with chronic constriction injury, 2-, composite 2/15-, and 100-Hz EA increased hippocampal M1 muscarinic and β2 nicotinic receptor expression, while the composite 2/15-Hz protocol additionally increased α4 nicotinic receptor mRNA. Intra-hippocampal blockade of M1 muscarinic or α4β2 nicotinic receptors abolished EA-induced analgesia, supporting the involvement of hippocampal cholinergic signaling (Chen S. P. et al., 2016).

Purine and prostaglandin responses also differed between protocols. Compared with manual acupuncture, the composite 2/15-Hz EA protocol increased subcortical adenosine and cortical prostaglandin D₂, while adenosine immunoreactivity in the ventrolateral preoptic nucleus was higher than after 50-Hz EA (Chai et al., 2025). In mice with irritable bowel syndrome, 2-, 15-, and composite 2/15-Hz EA reduced hypothalamic corticotropin-releasing factor and CRF receptor 1 and reversed the reduction in CRF receptor 2. The composite 2/15-Hz protocol produced a greater reduction in CRF receptor 1 and a greater increase in CRF receptor 2 than either single-frequency condition (Xiang et al., 2026).

In the hippocampal CA1 region of a methamphetamine-induced neurotoxicity model, 2-Hz EA preserved blood–brain barrier integrity and reduced neuronal injury, microglial activation, and pro-inflammatory signaling, whereas 10-Hz EA did not produce significant changes in these outcomes (Zhang et al., 2025).

##### 3.2.2.2 Neuronal activity and circuit recruitment

In the ventrobasal thalamus, both 2- and 100-Hz stimulation inhibited neuronal responses to colorectal distension. When stimulation was delivered within the center of the recorded neuron’s somatic receptive field, inhibition was greater at 100 Hz than at 2 Hz, whereas stimulation at ipsilateral or contralateral ST36 produced weaker inhibition (Zhang et al., 2009).

Frequency-related differences were also observed in neural oscillations. The composite alternating 2/15-Hz protocol was associated primarily with changes in δ-band activity, whereas 50-Hz EA altered α-, β-, and θ-band activity (Chai et al., 2025).

Studies using regional inactivation and electrical stimulation provide more direct evidence for the brain regions involved in EA responses. In chronic neuropathic pain, inactivation of the infralimbic medial prefrontal cortex blocked the responses to both 2-Hz and composite 2/100-Hz EA, whereas electrical stimulation of this region enhanced them. The infralimbic cortex therefore contributed to a response shared by single low-frequency and composite alternating-frequency stimulation (Leite Ferreira et al., 2022).

##### 3.2.2.3 Neuroplasticity and structural remodeling

Repeated stimulation introduced frequency-related transcriptional adaptations. Comparison of repeated 2- and 100-Hz EA over six days showed that 100 Hz reduced κ-opioid receptor mRNA in the striatum and amygdala. Repeated 2-Hz EA decreased expression in the periaqueductal gray and striatum but produced increased or biphasic changes in the amygdala. Opioid adaptation during repeated EA therefore depended on both frequency and brain region (Wang et al., 1998).

Frequency also affected trophic signaling and electrophysiological plasticity. In the post-stroke dysphagia model, 2-Hz EA increased brain-derived neurotrophic factor in the contralateral motor cortex (Yao et al., 2020). In an Alzheimer’s disease model, both 2- and 50-Hz EA increased the magnitude of hippocampal long-term potentiation and long-term depression, with larger changes after 50-Hz stimulation (Li et al., 2016). In cerebral ischemia, 5- and 50-Hz EA increased brain-derived neurotrophic factor- and nerve growth factor-positive cells in the ipsilesional hippocampal CA3 region, with greater increases after 50 Hz (Duan et al., 2018). Structural synaptic responses showed a similar intermediate-frequency pattern. In another Alzheimer’s disease model, 2-, 30-, and 50-Hz EA increased synapse number and density in the hippocampal CA1 region, with greater preservation after 50 Hz than after the 2- and 30-Hz protocols (Wang et al., 2016). Frequency-related structural effects were not confined to hippocampal synapses. Following middle cerebral artery occlusion, 15- and 30-Hz EA increased glial fibrillary acidic protein expression and preserved astrocytic ultrastructure at the infarct border more effectively than 100-Hz stimulation. Astrocytic nuclei, intracellular organelles, and perisynaptic structures were better preserved in the 15- and 30-Hz groups, which also showed less severe neuronal ultrastructural damage (Xiao et al., 2013). Across these direct comparisons, the frequency associated with the largest response varied among brain regions, experimental models, and measured endpoints.

### 3.3 Single-protocol evidence from needle-based EA

#### 3.3.1 Human brain networks

Repeated 2-Hz EA in patients with carpal tunnel syndrome altered primary somatosensory cortical connectivity with the thalamic pulvinar and anterior hippocampus, and the extent of cortical-network normalization was associated with later clinical improvement (Fisher et al., 2021). Low-frequency cerebral responses have also been examined in affective and post-stroke disorders. In first-episode major depressive disorder, 1-Hz EA increased the amplitude of low-frequency fluctuations in the left middle temporal gyrus and posterior cerebellum. Connectivity increased between the left middle temporal gyrus and the superior frontal gyrus, middle frontal gyrus, and hippocampus, while connectivity between the posterior cerebellum and calcarine cortex decreased (Wang et al., 2024). In patients with stroke-related sleep disorders, 2-Hz scalp EA reduced activity in the right medial superior frontal gyrus, precuneus, and posterior cingulate cortex and increased activity in the left angular gyrus, inferior parietal lobule, and middle occipital gyrus (Boyang et al., 2024).

Defined alternating-frequency protocols have been associated with changes in auditory, interoceptive, and brain–gut networks. In patients with subjective tinnitus, 2/50-Hz EA reduced insula-centered functional connectivity with the right middle frontal gyrus, left superior frontal gyrus, and right paracentral lobule (Zha et al., 2024). Under the same frequency pattern, anterior cingulate connectivity with frontal, temporal, and thalamic regions also changed after treatment (Zhang et al., 2024). In patients with Crohn’s disease, repeated 2/100-Hz EA increased functional connectivity between the bilateral hippocampus and the anterior midcingulate cortex and insula (Bao et al., 2017).

#### 3.3.2 Animal neurobiological evidence

Functional imaging under a defined low-frequency protocol provides network-level evidence. In the 5xFAD mouse model of Alzheimer’s disease, 2-Hz EA increased hippocampal functional connectivity and strengthened connections between limbic and neocortical regions, including hippocampal–insular connectivity (Xu et al., 2024). In rats with remifentanil-induced hyperalgesia, 2-Hz EA reduced spontaneous and stimulus-evoked neuronal hyperactivity and suppressed enhanced θ-band local-field-potential oscillations across the ventral posterolateral thalamic nucleus, primary somatosensory cortex, insular cortex, and anterior cingulate cortex (Cai et al., 2025).

Single-protocol studies also examined opioid signaling during repeated EA. Under a defined 2-Hz protocol, repeated EA increased enkephalin content and preproenkephalin mRNA in the nucleus accumbens. Its behavioral effect was reversed by intracerebroventricular μ- and δ-opioid receptor antagonists but not by a κ-opioid receptor antagonist (Liang et al., 2010). In morphine-dependent rats, repeated 100-Hz EA restored reduced preprodynorphin mRNA in the periaqueductal gray and hypothalamus and reversed the associated increase in phosphorylated cyclic adenosine monophosphate response element-binding protein (Wang et al., 2011).

Cell-type-specific recordings have identified local cortical microcircuits engaged by low-frequency stimulation. In mice with neuropathic pain, 2-Hz EA suppressed excitatory pyramidal neurons and vasoactive intestinal peptide-positive interneurons in the primary somatosensory cortex while increasing the activity of somatostatin-positive inhibitory interneurons (Wei et al., 2021). In mice exposed to chronic unpredictable stress, 2-Hz EA reduced the elevated excitatory-to-inhibitory ratio of pyramidal neurons in the dorsolateral and medial prefrontal cortices and ventral hippocampal CA1 region (Ma et al., 2025). These findings link the regional cortical response to changes in the balance between excitatory and inhibitory cell populations. Circuit-level evidence further extended from local cortical microcircuits to the rostral anterior cingulate cortex–ventrolateral periaqueductal gray pathway. Under a defined 2-Hz protocol, fiber-optic calcium imaging showed increased activity of CaMKII-positive neurons in the rACC after EA. In the same study, chemogenetic activation of the rACC–vlPAG projection reversed the behavioral effects of 2-Hz EA (Zhang et al., 2026).

Single-protocol follow-up experiments also examined selected composite protocols. In the blood–brain barrier study described above, follow-up assessment of the selected composite 2/100-Hz condition showed increased cortical perfusion and cortical c-Fos expression (Zhang et al., 2020). After a comparative protocol-screening stage in traumatic brain injury, detailed analysis of the selected composite 2/100-Hz protocol showed suppression of cerebral oxidative stress and the NF-κB/COX2 pathway, a shift in microglial markers toward an anti-inflammatory profile, and reduced neuronal apoptosis (Zhang et al., 2023).

### 3.4 Complementary TEAS evidence

Non-invasive transcutaneous electrical acupoint stimulation provides complementary evidence and is presented separately from needle-based EA (Supplementary Table S5). In a composite between-group TEAS comparison of 2 Hz with a 0.6-ms pulse width and 100 Hz with a 0.2-ms pulse width, analgesia under both protocols was positively correlated with activity in the bilateral secondary somatosensory cortices and insula, contralateral anterior cingulate cortex, and thalamus. The 2-Hz response additionally involved the contralateral primary motor cortex, supplementary motor area, and ipsilateral superior temporal gyrus, whereas the 100-Hz response involved the contralateral inferior parietal lobule, ipsilateral anterior cingulate cortex, nucleus accumbens, and pons (Zhang et al., 2003).

Perfusion fMRI showed a biphasic cerebral blood-flow response at both 2 and 100 Hz. The 2-Hz condition involved broader cortical and default-mode regions, whereas 100-Hz stimulation preferentially involved the ventral striatum and dorsal anterior cingulate cortex (Jiang et al., 2014). Post-stimulation EEG source localization showed that 2.5 pulses per second increased theta activity in the parahippocampal gyrus, whereas 10 pulses per second reduced alpha activity successively in the inferior parietal gyrus, postcentral gyrus, and insula (Lopes Alves et al., 2025).

## 4 Current intensity

Current intensity has been defined differently between human and animal EA studies. Human experiments commonly adjust stimulation according to individual sensory tolerance or motor responses, whereas animal experiments more often use fixed current amplitudes. The available brain-level evidence includes cerebral responses during and after stimulation, brainstem electrophysiology, cerebral perfusion, and autonomic circuit recruitment.

### 4.1 Human evidence

Human neuroimaging studies indicate that stimulation intensity alters both the magnitude and spatial distribution of cerebral responses to EA (Xiong et al., 2019; Xiong et al., 2020). During experimentally induced heat pain, minimal intensity was set at the first perceptible electrical sensation, whereas optimized intensity was increased to the strongest tolerable level without sharp pain or discomfort. Minimal-intensity stimulation activated the caudate and frontal regions and decreased signals in the primary and secondary somatosensory cortices and posterior cingulate cortex. Optimized-intensity EA produced more extensive signal decreases involving the secondary somatosensory cortex, dorsal anterior cingulate cortex, premotor cortex, amygdala, parietal cortex, and brainstem raphe or reticular regions. Significant intensity-related interactions were detected in the secondary somatosensory cortex, insula, anterior cingulate cortex, and premotor cortex, while heat-evoked thalamic activation was almost completely suppressed during optimized-intensity EA but only partially reduced during minimal-intensity stimulation (Shukla et al., 2011).

Intensity-related differences were also observed at ST37. In a crossover fMRI experiment, the mean amplitudes were 0.65 ± 0.32 and 1.61 ± 0.38 mA. Low-intensity EA mainly activated the left insula and decreased right precentral activity, whereas high-intensity EA recruited the bilateral middle temporal gyri, right insula, and right inferior parietal lobule and produced broader deactivation across medial prefrontal, sensorimotor, parietal, and inferior temporal regions (Xiong et al., 2020). Post-stimulation differences were examined in a separate resting-state fMRI study, in which the mean current amplitudes were 0.50 ± 0.02 mA in the low-intensity group and 1.10 ± 0.10 mA in the high-intensity group. Post-stimulation changes involved the precentral and postcentral gyri, superior and inferior parietal lobules, superior, middle, and inferior temporal gyri, insula, thalamus, anterior and posterior cingulate cortices, middle frontal gyrus, orbitofrontal cortex, and several occipital regions. Compared with the pre-stimulation resting state, signal amplitude decreased in most of these regions after low-intensity EA but increased in most regions after high-intensity EA (Xiong et al., 2019). During the second post-stimulation resting period, signal amplitudes in all identified regions were higher in the high-intensity group than in the low-intensity group. Threshold-defined intensity was also examined using the blink reflex, whose late bilateral R2 component depends on polysynaptic trigemino-facial brainstem circuits (Kofler et al., 2024). Both sensory- and motion-threshold EA shortened R2 latency relative to baseline in patients with peripheral facial paralysis, with a larger reduction at motion threshold (Huang et al., 2025).

### 4.2 Experimental evidence

Fixed-current experiments demonstrated a graded, non-linear intensity response in neurons of the medullary subnucleus reticularis dorsalis. During colorectal distension, 0.5–1 mA produced little change, whereas currents from 2 to 6 mA progressively increased spontaneous discharge and strengthened the inhibition of distension-evoked nociceptive firing; stimulation above 6 mA produced little additional neuronal response (Yu et al., 2018). A subsequent study in rats with inflammatory visceral hypersensitivity identified a similar range, with 2-, 3-, and 6-mA EA producing progressively greater inhibition of nociceptive firing and recruiting a larger proportion of responsive neurons. These findings indicate that medullary nociceptive processing shows an intensity-dependent response within approximately 2–6 mA, followed by a plateau at higher currents (Li et al., 2025). Current amplitude also altered the perfusion of ischemic brain tissue. During middle cerebral artery occlusion, currents below approximately 0.6 mA produced little change, whereas 0.6–1.0 mA generated a graded increase in perfusion; at 1.0 mA, cerebral blood flow more than doubled, and 1.2 mA produced no further increase. The same effective current range reduced infarct volume. This plateauing response links current amplitude to regional perfusion and structural injury within the ischemic middle cerebral artery territory (Zhou et al., 2011). Intensity dependence also extended to cortical neurovascular and neuronal responses. During 100-Hz trigeminal EA, 1-, 3-, and 5-mA stimulation produced non-linear changes in blood–brain barrier permeability and frontal cortical neuronal activation, with both responses peaking at 3 mA rather than increasing further at 5 mA. In the trigeminal representation of the primary somatosensory cortex, glutamatergic calcium-wave events increased at 3 and 5 mA relative to prestimulation values but not at 1 mA, and the difference between the 3- and 5-mA groups was not significant (Gong et al., 2022). A different intensity-related pattern emerged in autonomic circuits. At the hindlimb acupoint ST36, 0.5-mA EA induced c-Fos in cholinergic neurons of the dorsal motor nucleus of the vagus but not in spinal sympathetic preganglionic neurons, whereas 3.0-mA EA recruited the spinal sympathetic pathway (Liu et al., 2020). Low-intensity stimulation at ST36 and high-intensity stimulation at the abdominal acupoint Tianshu (ST25) recruited the vagal–adrenal and spinal sympathetic pathways, respectively, but because both stimulation site and current intensity changed, this was a composite site–intensity comparison (Liu et al., 2020).

## 5 Other stimulation characteristics

Beyond frequency and current intensity, direct brain-level comparisons have mainly examined waveform and stimulation duration. These parameters alter the temporal arrangement and cumulative exposure of electrical pulses and have been associated with neurovascular, neuroimmune, neurochemical, and structural responses in the brain.

### 5.1 Waveform

Waveform changes how pulses are arranged over time within a stimulation session. Available brain-level studies have compared continuous, intermittent, and alternating protocols, primarily in models of cerebral injury.

In sepsis-associated brain injury, continuous 2-Hz, intermittent 2/0-Hz, and dilatational 2/15-Hz EA were compared with current intensity and duration matched across groups. The 2/15-Hz dilatational protocol produced the largest reductions in cerebral edema and blood–brain barrier leakage and the strongest preservation of hippocampal neurons, together with greater attenuation of hippocampal oxidative stress, microglial activation, inflammatory signaling, and apoptosis (Chen Y. et al., 2016). Waveform-related differences were also detected after cerebral ischemia–reperfusion injury. Continuous-wave EA was delivered at 15 Hz, discontinuous-wave EA used 15-Hz trains separated by intervals of approximately 1.5 s, and dense–disperse EA alternated between 3 and 15 Hz. All three protocols reduced cerebral infarct volume and suppressed cortical inflammatory and pyroptosis-related responses. Compared with continuous-wave stimulation, the dense–disperse protocol produced greater reductions in inflammatory cytokines and several pyroptosis-related markers; selected caspase-1- and ASC-related measures were also lower than after discontinuous stimulation (Yu et al., 2026). In these two brain-injury models, temporally alternating protocols produced larger neurovascular and neuroimmune changes than continuous stimulation. These comparisons represent composite temporal protocols because component frequency and train structure also differed.

### 5.2 Duration

Stimulation duration affected several brain-level processes, but the temporal profile differed across blood–brain barrier permeability, cerebral perfusion, neurochemical signaling, and structural tissue injury. A short-duration experiment examined blood–brain barrier permeability after EA at GV20 and GV26 in healthy rats and rats recovering from middle cerebral artery occlusion. Blood–brain barrier permeability was highest after 5 min in healthy rats and after 8 min in rats recovering from middle cerebral artery occlusion, and the selected 8-min protocol promoted the entry of administered nerve growth factor into brain tissue (Zhang et al., 2018). Longer stimulation produced a non-linear neurovascular response during acute cerebral ischemia. Across 5-, 15-, 30-, and 45-min protocols, infarct volume decreased progressively up to 30 min, but this reduction was no longer present after 45 min. Reperfusion also recovered after 5–30 min of EA but remained markedly impaired after 45 min. The reduction in infarct volume after 30 min was largely reversed by a δ-opioid receptor antagonist (Zhou et al., 2013b). Duration also influenced neurochemical and structural responses in injured brain tissue. In cerebral ischemia, 10-, 30-, and 60-min EA reduced glutamate, while the 60-min protocol additionally reduced aspartate (Zhang et al., 2007). After traumatic brain injury, 60-min EA, but not 30-min EA, significantly reduced cortical lesion volume, and the lesion was smaller after 60 than 30 min. The 60-min protocol also increased regional cerebral blood flow and reduced cortical neuronal apoptosis and TGIF expression relative to untreated injury (Chuang et al., 2013).

## 6 Acupoint selection

Acupoint selection adds an anatomical dimension to the electrical parameters described above. Direct comparisons indicate that changing the stimulation site can alter the spatial distribution of cerebral responses. In healthy volunteers, EA at GB37 and KI8 produced different whole-brain power patterns in the periaqueductal gray, occipital cortex, and temporal cortex, while differences in the sustained response involved the occipital cortex, dorsolateral and medial prefrontal cortices, anterior cingulate cortex, and insula (Liu et al., 2011).

Site-related responses were also evident within somatotopically organized sensorimotor networks. In patients with carpal tunnel syndrome, local EA at PC7 and TE5 produced greater activation in the insula and secondary somatosensory cortex and greater deactivation in the ipsilateral primary somatosensory cortex and inferior temporal gyrus, whereas distal EA at contralateral SP6 and LR4 produced greater bilateral secondary somatosensory cortical activation and posterior cingulate cortical deactivation relative to sham EA (Maeda et al., 2013). After repeated treatment, local and distal acupuncture increased fractional anisotropy adjacent to the hand and leg representations of the ipsilesional primary somatosensory cortex, respectively (Maeda et al., 2017). During swallowing, EA at HT5 plus GB20 produced greater left primary motor cortical activation and stronger interhemispheric motor connectivity than HT5 alone and strengthened sensorimotor–premotor connectivity relative to GB20 alone (Fu et al., 2023).

Although ST36 and ST25 are both located on the Foot-Yangming Stomach Meridian, their effects on the nucleus of the solitary tract depended on the point–frequency combination: at ST36, 2-Hz EA produced the strongest excitation, whereas 100-Hz and 2/100-Hz EA produced only transient changes; at ST25, 2-Hz EA produced no significant excitation, whereas 100-Hz and 2/100-Hz EA reduced neuronal excitability and spike frequency, with the strongest inhibition at 100 Hz (Fang et al., 2017). Anatomical differences further distinguished the autonomic effects of these points. PROKR2-expressing sensory neurons densely innervated the deep fascia beneath ST36 but not ST25 and were required for 10-Hz, 0.5-mA stimulation at ST36 to activate hindbrain vagal efferents and adrenal catecholamine release, whereas 10-Hz, 3.0-mA stimulation at either site activated choline acetyltransferase-positive sympathetic preganglionic neurons in the spinal intermediolateral nuclei and recruited a PROKR2-independent spinal–sympathetic pathway (Liu et al., 2021). At ST25, this pathway further activated NPY-expressing splenic noradrenergic neurons and induced splenic noradrenaline release (Liu et al., 2020).

Point-specific effects also extended from autonomic circuits to cerebral neurochemistry and cortical injury. Under matched 2-Hz EA conditions, stimulation at ST36 increased adrenaline in the cerebral cortex and hippocampus and increased hippocampal arginine, whereas stimulation at PC6 reduced γ-aminobutyric acid and glycine in the cerebral cortex and hippocampus and reduced hippocampal glutamate (Lee et al., 2020). At GV26, 2-Hz EA reduced large-conductance Ca^2+^-activated K^+^ channel expression and pathological injury in the cerebral cortex, whereas the same stimulation at a nearby non-acupoint did not produce these changes (Wang et al., 2016).

Point location and laterality also modified neurovascular responses in cerebral ischemia. In rats with right middle cerebral artery occlusion, matched 5/20-Hz, 1.0-mA EA at GV20 and GV26 or at left, lesion-contralateral LI11 and PC6 increased ischemic cortical cerebral blood flow and reduced infarct volume, whereas stimulation at right LI11 and PC6 or left GB34 and SP6 did not significantly change cerebral blood flow or infarct volume (Zhou et al., 2013a).

EA at GV20 also produced a point-specific brain-response pattern. Compared with stimulation at a sham point, EA at GV20 increased regional homogeneity in the orbitofrontal cortex, middle cingulate cortex, precentral cortex, and precuneus, while decreasing it in the anterior cingulate cortex, supplementary motor area, thalamus, putamen, and cerebellum (Deng et al., 2016). Beyond these direct comparisons, reported applied protocols illustrate how GV20 has been paired with different points for different brain-related targets. In sepsis-associated brain injury, GV20 and ST36 formed a head–limb prescription, and EA pretreatment at this point pair reduced cerebral edema and blood–brain barrier leakage and attenuated hippocampal neuronal injury, microglial activation, inflammatory signaling, oxidative stress, and apoptosis, with the dilatational waveform producing the strongest effects (Chen Y. et al., 2016). For transient blood–brain barrier opening after cerebral ischemia, GV20 and GV26 formed a craniofacial Governor Vessel prescription; at 100 Hz and 2 mA, the increase in permeability was most notable after 8 min in rats recovering from middle cerebral artery occlusion, and the same protocol facilitated nerve growth factor entry into brain tissue without increasing brain water content (Zhang et al., 2018). A GV20–GV26-centered protocol was also used clinically for recovery of consciousness: in a randomized study of 90 patients with coma after severe traumatic brain injury, 14 days of 1/50-Hz EA improved Glasgow Coma Scale scores and arousal relative to routine medication, with outcomes comparable to naloxone treatment (Zhang et al., 2013).

The recurrent anatomical distribution of brain responses identified in direct comparative studies across the five focal intervention dimensions is summarized in Figure 3, with tags indicating whether the evidence concerned frequency, current intensity, waveform, stimulation duration, or acupoint selection.

**Figure 3:**
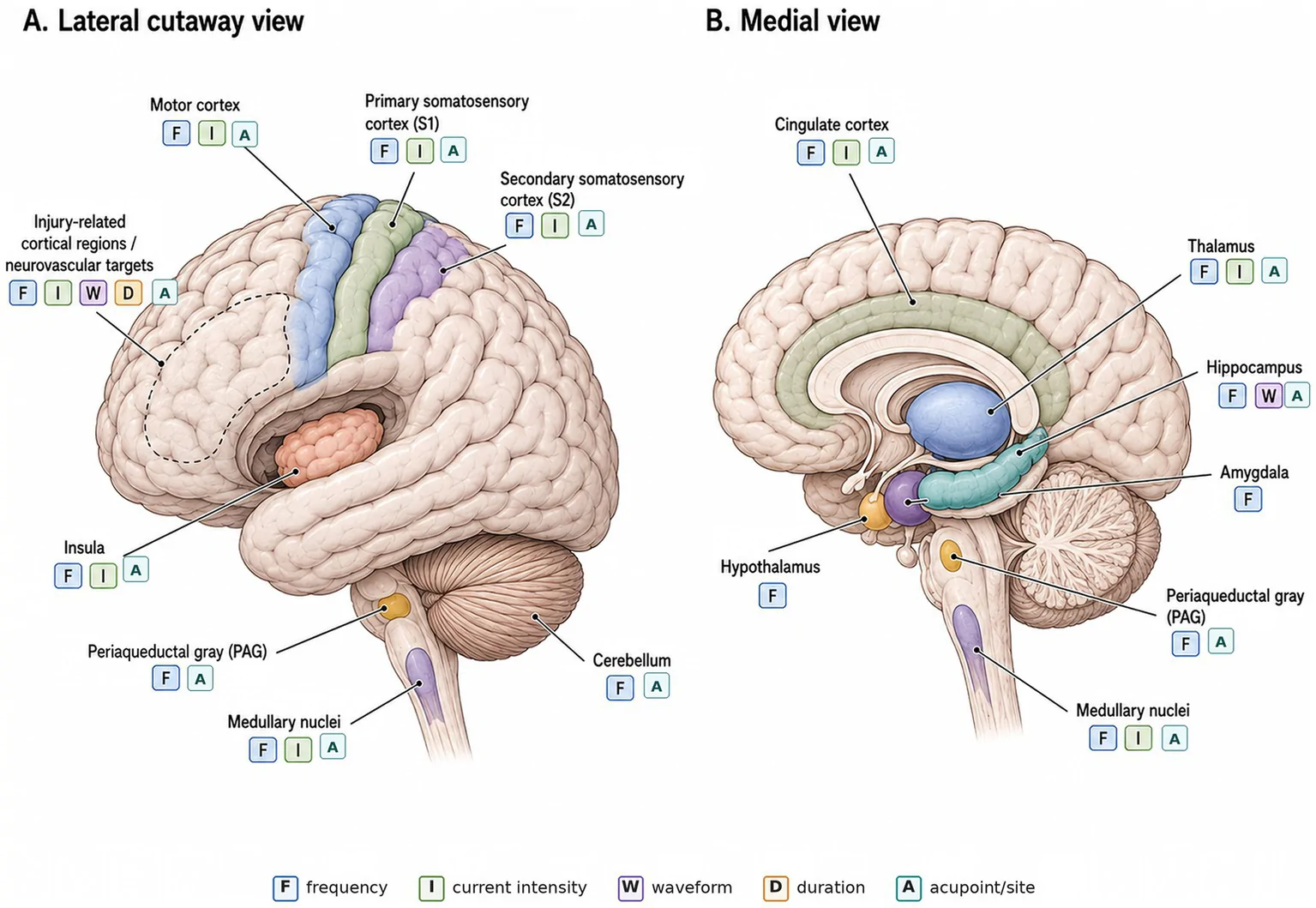
Anatomical distribution of brain regions investigated in direct comparative studies of needle-based electroacupuncture. Schematic lateral cutaway **(A)** and medial **(B)** views show the principal cortical and subcortical regions examined in studies comparing frequency, current intensity, waveform, stimulation duration, or acupoint/site. Colored labels indicate the focal intervention dimensions for which direct comparative evidence was identified at each region: F, frequency; I, current intensity; W, waveform; D, stimulation duration; and A, acupoint/site. Multiple labels associated with one structure indicate that more than one intervention dimension has been examined in that region. The dashed area marks grouped injury-related cortical regions or neurovascular targets rather than a single anatomically discrete structure. This schematic depicts anatomical coverage only and does not indicate response direction or magnitude, study counts for individual regions, evidence strength, or precise stereotaxic boundaries. EA, electroacupuncture; S1, primary somatosensory cortex; S2, secondary somatosensory cortex; PAG, periaqueductal gray.

## 7 Discussion

### 7.1 Response patterns

The available evidence indicates that EA parameters shape the organization of brain responses rather than generating completely separate cerebral maps. Across direct comparative human neuroimaging studies, different frequencies repeatedly involved overlapping somatosensory, insular, cingulate, thalamic, limbic, and motor systems, while altering the spatial extent and relative weighting of activity within these systems. Animal studies similarly identified frequency-related recruitment across the cerebral cortex, thalamus, hypothalamus, midbrain, and medulla. Because spatial recruitment and neurochemical, neurovascular, and plasticity-related outcomes were assessed using different methods, the extent of regional recruitment alone does not indicate the overall magnitude of the central response.

Within the direct comparative evidence, several frequency-related tendencies can nevertheless be identified. Low-frequency stimulation often recruited broader cortical, thalamic, and brainstem populations and was repeatedly associated with somatosensory, prefrontal, cingulate, and descending modulatory circuits. High-frequency stimulation was more prominent in dynorphin-related signaling and selected brainstem and thalamic responses. Intermediate frequencies produced larger changes in several hypothalamic, striatal, and hippocampal measures, including monoaminergic, excitatory–inhibitory, metabolic, trophic, and synaptic responses. Alternating-frequency protocols were prominent in selected cerebral perfusion, blood–brain barrier, cholinergic, stress-related, and neuroimmune outcomes. These tendencies depended on the brain region, pathological model, stimulation site, and measured endpoint rather than representing fixed frequency signatures.

Single-protocol evidence extended these findings from regional activity to functional networks, local microcircuits, receptor systems, cell populations, and defined neural projections under selected needle-based EA conditions. Complementary TEAS studies also identified overlapping and frequency-related cerebral responses, but were considered separately because transcutaneous and needle-based stimulation differ in the electrode–tissue interface, stimulation depth, and afferent recruitment.

Frequency therefore appears to select among regional, neurochemical, electrophysiological, neurovascular, and plasticity-related response modes rather than uniformly amplifying brain activity. Apparent response peaks at 15, 50, or 60 Hz depended on the tested brain region, pathological model, and endpoint. The available heterogeneous evidence therefore does not establish a universally optimal frequency.

### 7.2 Parameter interactions

Current intensity showed a similarly non-linear relationship with central responses. Human fMRI studies found different spatial patterns under minimal, optimized, low-, and high-intensity conditions, while experimental studies identified recruitment thresholds and response plateaus in medullary neurons, cerebral perfusion, blood–brain barrier permeability, and cortical neuronal activity. These findings indicate that increasing current amplitude does not necessarily produce a proportionally larger brain response. Different neuronal and neurovascular processes may instead have distinct activation thresholds and saturation ranges.

Intensity was not operationalized consistently across studies. Human experiments usually used perceptual or motor thresholds, whereas animal studies reported fixed current amplitudes. Moreover, stimulation site and intensity varied together in the autonomic experiments, so the resulting pathway differences reflected both electrical intensity and anatomical site. The recruitment of vagal or sympathetic pathways is more appropriately interpreted as the product of an interaction among intensity, tissue depth, stimulation site, and the sensory afferents available at that site.

Acupoint selection adds a further anatomical dimension to these interactions. Direct site comparisons showed that changing the stimulated point altered regional cerebral recruitment, somatotopic sensorimotor organization, neurochemical responses, and neurovascular regulation. The different responses produced at ST36 and ST25 further indicate that points on the same meridian do not necessarily recruit the same brainstem or autonomic pathways. The effects of frequency and intensity therefore depend partly on the anatomical and afferent characteristics of the selected site.

A comparable interpretive problem affects waveform studies. Continuous, intermittent, and dense–disperse protocols differ not only in temporal arrangement but often also in component frequency and train structure. The larger neurovascular or neuroimmune changes associated with alternating waveforms may therefore reflect the combined effects of frequency composition, transitions between frequencies, interruptions between pulse trains, and total pulse exposure. In this context, a dense–disperse protocol represents a composite stimulation pattern rather than a waveform variable isolated from frequency.

Duration is also inseparable from cumulative electrical exposure. Extending the stimulation period increases the total number of delivered pulses and may change the balance among initial neural recruitment, adaptation, vascular regulation, and recovery. The reviewed studies showed that longer stimulation did not produce uniformly larger brain responses: blood–brain barrier opening peaked after a short exposure, ischemic perfusion and structural responses were most evident at an intermediate duration, and a longer protocol was required for the clearest cortical response after traumatic brain injury. Duration therefore showed endpoint- and model-dependent response ranges rather than a universal optimum.

Together, frequency, current intensity, waveform, duration, and acupoint/site form a multidimensional intervention. Their biological effects cannot always be separated by examining one nominal setting in isolation, particularly when alternating-frequency or waveform protocols change several electrical characteristics simultaneously.

### 7.3 Evidence interpretation

EA-related brain responses were assessed during stimulation, immediately afterward, and after repeated treatment, but these time points represent different biological processes. Acute measurements primarily capture immediate afferent, neuronal, oscillatory, and neurovascular responses, whereas delayed and repeated-treatment measurements can reflect transcriptional adaptation, network reorganization, and structural plasticity. Parameter effects obtained at different time points should therefore not be treated as directly equivalent. A parameter may be prominent during acute stimulation but show a different pattern during post-stimulation or repeated-treatment assessment.

Differences among measurement methods further affect the interpretation of the evidence. BOLD fMRI and perfusion imaging characterize distributed haemodynamic responses, EEG and local-field potentials capture temporal activity, c-Fos-based methods identify recruited cell populations, and microdialysis or molecular assays assess specific neurochemical processes. Calcium recording provides cell-type- or circuit-level temporal information, whereas pharmacological blockade, regional inactivation, and chemogenetic manipulation can test whether a region or pathway is required for the observed response. Agreement across these methods would provide stronger evidence than any single marker, but few studies have compared multiple EA parameters across more than one level of neural organization.

Statistical significance and direction of change should also be distinguished from evidence strength. A significant fMRI contrast, a difference in c-Fos expression, a neurochemical change, and a causal circuit-manipulation result describe different levels of neural organization and inference. Their evidential value depends on the study design, sample size, control condition, correction for multiple comparisons, randomization or blinding, and whether the experiment demonstrates association or causal involvement.

The distinction between circuit involvement and parameter specificity is equally important. Pharmacological blockade and circuit manipulation support the involvement of hippocampal cholinergic receptors, the infralimbic medial prefrontal cortex, local cortical microcircuits, and the rACC–vlPAG pathway in responses to the tested EA protocols. When detailed mechanistic experiments are performed under only one selected protocol, however, they establish involvement under that condition rather than demonstrating that the mechanism is unique to that frequency. Parameter specificity requires direct comparison of multiple parameter conditions while the remaining stimulation characteristics are held constant.

Human neuroimaging findings are additionally influenced by the conditions under which stimulation is experienced. Expectation, perceived sensory intensity, subjective deqi, control-condition design, and participant blinding can influence BOLD and functional-connectivity responses. These variables should be measured and reported when differences between stimulation conditions are interpreted as parameter-related neural effects.

Translation between human and animal evidence remains incomplete. Human studies predominantly characterize regional activity, functional connectivity, perfusion, or brainstem neurophysiology, whereas animal experiments allow invasive recording, tissue analysis, and circuit manipulation. Much of the waveform and duration evidence comes from models of cerebral ischemia, traumatic brain injury, or systemic inflammation. Altered blood flow, barrier integrity, neuronal excitability, and immune activity in these models can modify the response to stimulation. These findings are informative for the pathological brain, but their applicability to healthy individuals and unrelated chronic neurological conditions requires direct examination.

The evidence base is uneven across the focal intervention dimensions. Frequency has the largest and most methodologically diverse literature, although studies remain heterogeneous in species, disease model, stimulation site, frequency range, endpoint, and assessment time point. Current intensity has been examined in fewer studies, while waveform and duration evidence is restricted largely to animal injury models. Direct brain-level comparative evidence for pulse width remains insufficient. Acupoint-selection studies demonstrate site-related cerebral and circuit responses, but direct comparisons among clinically relevant point prescriptions remain uncommon.

### 7.4 Translational implications and research priorities

The reviewed evidence has direct implications for the design of human electroacupuncture protocols, but parameter values cannot be transferred from animals to patients on a one-to-one basis. Fixed currents used in animal studies interact with body size, tissue depth, stimulation site, needle geometry, and the afferent fibers accessible at each site, whereas human studies usually titrate current according to sensory tolerance or motor responses. Future human studies should therefore report both the absolute current amplitude and its relation to individual sensory or motor thresholds, together with needle depth, pulse width, waveform, duty cycle, and stimulation site. Animal findings are most useful for identifying response principles, including thresholds, plateaus, and region-specific recruitment, rather than directly transferable clinical doses.

Parameter and acupoint selection should be target-oriented rather than convention-driven. The current evidence does not support a single optimal frequency, intensity, or point prescription across all brain systems. The preferred protocol may depend on whether the intended target is sensorimotor-network recruitment, limbic and interoceptive processing, descending pain modulation, neurovascular regulation, neuroimmune signaling, or synaptic plasticity. The reviewed applications used GV20–ST36 for cerebral edema and hippocampal neuroinflammation, GV20–GV26-centered prescriptions for neurovascular regulation and recovery of consciousness, and local or distal limb points for somatotopically related sensorimotor networks. These applications illustrate how point selection has been matched to different therapeutic objectives, although few studies have directly compared clinically plausible prescriptions under the same electrical conditions.

Future studies of acupoint selection should compare alternative points or point combinations while matching frequency, current intensity, pulse width, waveform, duration, needle depth, and treatment schedule. Such comparisons are needed to determine whether the observed brain response is related to point location, laterality, point combination, tissue anatomy, or an interaction between site and electrical parameters. Brain-level outcomes should also be related to clinically relevant changes in consciousness, motor function, sensory function, or neurovascular status.

More generally, future parameter studies should vary one electrical dimension while holding the remaining stimulation conditions constant. Multilevel rather than two-level comparisons are needed to identify thresholds, plateaus, and non-linear response ranges. Complete reporting should include frequency, current amplitude, pulse width, waveform, train structure, duty cycle, switching interval, stimulation duration, total pulse exposure, needle depth, stimulation site, and number of treatment sessions. Direct comparative studies can first identify parameter- or site-related differences, followed by single-protocol recording or causal manipulation to examine the neural mechanisms engaged by the selected condition. This combination would support reproducible and mechanism-informed electroacupuncture protocol design.

## 8 Conclusion

EA parameters shape the relative recruitment of shared brain systems rather than assigning each setting to an entirely separate cerebral map. Frequency altered regional activity, network connectivity, neurochemical signaling, neuronal discharge and oscillations, neurovascular responses, and plasticity. Low-, intermediate-, high-, and alternating-frequency protocols each produced prominent responses in selected systems, but the available heterogeneous evidence does not establish a universally optimal frequency. Single-protocol evidence characterized receptors, cell populations, local microcircuits, and defined neural projections engaged under selected conditions, whereas parameter specificity rests on direct within-study comparisons. Complementary TEAS findings remain distinct from needle-based EA.

The evidence base is uneven. Frequency has the largest and most diverse literature, although studies vary widely in species, disease model, stimulation site, frequency range, endpoint, and assessment time point. Current intensity is supported by fewer studies and shows recruitment thresholds, non-linear response ranges, and plateaus. Waveform evidence is very limited and often involves concurrent differences in frequency composition and train structure, whereas duration evidence is also limited and strongly dependent on the model and endpoint. Direct brain-level comparative evidence for pulse width remains insufficient. Acupoint and stimulation-site comparisons indicate that point location, laterality, point combination, and local afferent anatomy can modify cerebral and circuit responses, although clinically relevant point prescriptions have rarely been compared directly under matched electrical conditions.

Together, frequency, current intensity, pulse width, waveform, and duration constitute a multidimensional electrical dose, while acupoint selection adds an anatomical dimension. Parameter and point selection should therefore be matched to the intended brain region, network, or biological process. Controlled comparisons, complete reporting of stimulation characteristics, cross-species normalization, and validation against clinically relevant outcomes are required for reproducible and mechanism-informed EA protocol design.

## References

[ref1] Bao C. Wang D. Liu P. Shi Y. Jin X. Wu L. . (2017). Effect of electro-acupuncture and moxibustion on brain connectivity in patients with crohn's disease: a resting-state fmri study. Front. Hum. Neurosci. 11:559. doi: 10.3389/fnhum.2017.00559, 29204113 PMC5698267

[ref2] Boyang Z. Yang Z. Liyuan F. Dan S. Lei H. E. Dan T. . (2024). A neural regulation mechanism of head electroacupuncture on brain network of patients with stroke related sleep disorders. J. Traditional Chinese Med. 44, 1268–1276. doi: 10.19852/j.cnki.jtcm.2024.06.011, 39617712 PMC11589561

[ref3] Cai S. Tian Y. Zhang Z. Zhao H. Cai J. Li M. . (2025). Contribution of thalamo-cortico-limbic circuits to remifentanil-induced hyperalgesia (RIH) and the analgesic effects of electroacupuncture on RIH. Brain Res. Bull. 232:111582. doi: 10.1016/j.brainresbull.2025.111582, 41083053

[ref4] Chai Q. Ma J. Shu J. Wang H. Xu Y. Zhu S. . (2025). Effects of different frequencies of electroacupuncture stimulation on the electroencephalogram and intracranial levels of prostaglandin d(2) and adenosine in rats under propofol anesthesia. Acupunct. Med. 43, 174–180. doi: 10.1177/09645284251344329, 40437703

[ref5] Chang F. Tsai H. Yu M. Yi P. Lin J. (2004). The central serotonergic system mediates the analgesic effect of electroacupuncture on zusanli (st36) acupoints. J. Biomed. Sci. 11, 179–185. doi: 10.1007/BF02256561, 14966368

[ref6] Chen S. P. Kan Y. Zhang J. L. Wang J. Y. Gao Y. H. Qiao L. N. . (2016). Involvement of hippocampal acetylcholinergic receptors in electroacupuncture analgesia in neuropathic pain rats. Behav. Brain Func. 12:13. doi: 10.1186/s12993-016-0096-x, 27068709 PMC4828850

[ref7] Chen Y. Lei Y. Mo L. Li J. Wang M. Wei J. . (2016). Electroacupuncture pretreatment with different waveforms prevents brain injury in rats subjected to cecal ligation and puncture via inhibiting microglial activation, and attenuating inflammation, oxidative stress and apoptosis. Brain Res. Bull. 127, 248–259. doi: 10.1016/j.brainresbull.2016.10.009, 27771396

[ref8] Cheng L. Ding M. Xiong C. Zhou M. Qiu Z. Wang Q. (2012). Effects of electroacupuncture of different frequencies on the release profile of endogenous opioid peptides in the central nerve system of goats. Evidence-Based Complem. Alter. Med. 2012:476457. doi: 10.1155/2012/476457, 23133494 PMC3486625

[ref9] Chuang C. H. Hsu Y. C. Wang C. C. Hu C. Kuo J. R. (2013). Cerebral blood flow and apoptosis-associated factor with electroacupuncture in a traumatic brain injury rat model. Acupun. Med. 31, 395–403. doi: 10.1136/acupmed-2013-010406, 24055977

[ref10] Deng D. Duan G. Liao H. Liu Y. Wang G. Liu H. . (2016). Changes in regional brain homogeneity induced by electro-acupuncture stimulation at the baihui acupoint in healthy subjects: a functional magnetic resonance imaging study. J. Alter. Complement. Med. 22, 794–799. doi: 10.1089/acm.2015.0286, 27438091

[ref11] Duan X. Zhang L. Yu J. Wei W. Liu X. Xu F. . (2018). The effect of different frequencies of electroacupuncture on bdnf and ngf expression in the hippocampal ca3 area of the ischemic hemisphere in cerebral ischemic rats. Neuropsychiatr. Dis. Treat. 14, 2689–2696. doi: 10.2147/NDT.S183436, 30349267 PMC6187974

[ref12] Fang J. Du J. Shao X. Fang J. Liu Z. (2017). Effect of electroacupuncture on the nts is modulated primarily by acupuncture point selection and stimulation frequency in normal rats. BMC Complement. Altern. Med. 17:182. doi: 10.1186/s12906-017-1690-7, 28359336 PMC5374564

[ref13] Fisher H. Sclocco R. Maeda Y. Kim J. Malatesta C. Gerber J. . (2021). S1 brain connectivity in carpal tunnel syndrome underlies median nerve and functional improvement following electro-acupuncture. Front. Neurol. 12:754670. doi: 10.3389/fneur.2021.754670, 34777225 PMC8578723

[ref14] Fu X. Li H. Yang W. Li X. Lu L. Guo H. . (2023). Electroacupuncture at ht5 + gb20 produces stronger activation effect on swallowing cortex and muscle than single points. Heliyon 9:e21922. doi: 10.1016/j.heliyon.2023.e21922, 38034817 PMC10682618

[ref15] Gong P. Zhang S. Ren L. Zhang J. Zhao Y. Mao X. . (2022). Electroacupuncture of the trigeminal nerve causes n-methyl-d-aspartate receptors to mediate blood-brain barrier opening and induces neuronal excitatory changes. Front. Cell. Neurosci. 16:1020644. doi: 10.3389/fncel.2022.1020644, 36313622 PMC9606778

[ref16] Guo H. F. Wang X. M. Tian J. H. Huo Y. P. Han J. S. (1997). 2 hz and 100 hz electroacupuncture accelerate the expression of genes encoding three opioid peptides in the rat brain. Sheng Li Xue Bao 49, 121–127. 9812845

[ref17] Guo Z. Wei N. Ye R. Sun T. Qiu S. Shao X. (2024). Map activation of various brain regions using different frequencies of electroacupuncture st36, utilizing the foscreer strategy. Acupunct. Herbal Med. 4, 386–398. doi: 10.1097/HM9.0000000000000106

[ref18] He X. Zhu F. Duan C. Sun S. (2026). Effect of electroacupuncture at different frequencies on motor function in patients with post-stroke hemiplegia based on bold-fmri. Zhongguo Zhen Jiu 46, 695–703. Chinese. doi: 10.13703/j.0255-2930.20250422-k0004 42116772

[ref19] Huang C. Lin Y. Lee D. Hsieh C. (2019). Electroacupuncture relieves cci-induced neuropathic pain involving excitatory and inhibitory neurotransmitters. Evidence-Based Complem. Alter. Med. 2019:6784735. doi: 10.1155/2019/6784735, 31772598 PMC6854981

[ref20] Huang J. Zhong C. Zheng Y. Li W. Liang X. Li S. . (2025). Therapeutic effect of electroacupuncture at different intensities on peripheral facial paralysis and its influence on brainstem excitability. Zhen Ci Yan Jiu 50, 1449–1455. doi: 10.13702/j.1000-0607.20240877 41443919

[ref21] Huo M. Wang Q. Zhao R. Lin Y. Feng B. (2023). Effect of electroacupuncture at different frequencies on brain insulin signaling transduction pathway in Alzheimer's disease mice. Zhongguo Zhen Jiu 43, 60–66. doi: 10.13703/j.0255-2930.20220514-0005 36633241

[ref22] Jang-Hern L. Beitz A. J. (1993). The distribution of brain-stem and spinal cord nuclei associated with different frequencies of electroacupuncture analgesia. Pain 52, 11–28. doi: 10.1016/0304-3959(93)90109-3, 8446432

[ref23] Jiang Y. Liu J. Liu J. Han J. Wang X. Cui C. (2014). Cerebral blood flow-based evidence for mechanisms of low- versus high-frequency transcutaneous electric acupoint stimulation analgesia: a perfusion fmri study in humans. Neuroscience 268, 180–193. doi: 10.1016/j.neuroscience.2014.03.019, 24657460

[ref24] Kofler M. Hallett M. Iannetti G. D. Versace V. Ellrich J. Téllez M. J. . (2024). The blink reflex and its modulation - part 1: physiological mechanisms. Clinical Neurophysiol. 160, 130–152. doi: 10.1016/j.clinph.2023.11.015, 38102022 PMC10978309

[ref25] Lee D. Jiu Y. Hsieh C. (2020). Electroacupuncture at zusanli and at neiguan characterized point specificity in the brain by metabolomic analysis. Sci. Rep. 10:10717. doi: 10.1038/s41598-020-67766-0, 32612281 PMC7329888

[ref26] Leite Ferreira L. Pereira Generoso L. Medeiros A. C. de Medeiros P. Leonardo De Freitas R. Lourenço Da Silva M. . (2022). Infralimbic medial prefrontal cortex alters electroacupuncture effect in animals with neuropathic chronic pain. Behav. Brain Res. 424:113803. doi: 10.1016/j.bbr.2022.113803, 35189173

[ref27] Li W. Kong L. Wang H. Shen F. Wang Y. Zhou H. . (2016). High-frequency electroacupuncture evidently reinforces hippocampal synaptic transmission in alzheimer's disease rats. Neural Regen. Res. 11, 801–806. doi: 10.4103/1673-5374.182708, 27335565 PMC4904472

[ref28] Li Y. Sun X. Zhang Z. Wang X. Yuan M. Su Y. . (2025). Analgesic effects of electroacupuncture at different intensities on visceral pain in rats. Zhen Ci Yan Jiu 50, 243–250. doi: 10.13702/j.1000-0607.20241090 40103375

[ref29] Liang J. Ping X. Li Y. Ma Y. Wu L. Han J. . (2010). Morphine-induced conditioned place preference in rats is inhibited by electroacupuncture at 2 hz: role of enkephalin in the nucleus accumbens. Neuropharmacology 58, 233–240. doi: 10.1016/j.neuropharm.2009.07.007, 19596017

[ref30] Liu S. Wang Z. Su Y. Qi L. Yang W. Fu M. . (2021). A neuroanatomical basis for electroacupuncture to drive the vagal-adrenal axis. Nature 598, 641–645. doi: 10.1038/s41586-021-04001-4, 34646018 PMC9178665

[ref31] Liu S. Wang Z. Su Y. Ray R. S. Jing X. Wang Y. . (2020). Somatotopic organization and intensity dependence in driving distinct NPY-expressing sympathetic pathways by electroacupuncture. Neuron 108, 436–450.e7. doi: 10.1016/j.neuron.2020.07.015, 32791039 PMC7666081

[ref32] Liu P. Zhou G. Yang X. Liu J. Sun J. Dong M. . (2011). Power estimation predicts specific function action of acupuncture: an fmri study. Magn. Reson. Imaging 29, 1059–1064. doi: 10.1016/j.mri.2011.06.002, 21849241

[ref33] Lopes Alves R. Zortea M. Mayor D. Watson T. Steffert T. (2025). Effect of different frequencies of transcutaneous electrical acupoint stimulation (teas) on eeg source localization in healthy volunteers: a semi-randomized, placebo-controlled, crossover study. Brain Sci. 15:270. doi: 10.3390/brainsci15030270, 40149791 PMC11940437

[ref34] Ma Z. Huang Z. Chen J. Xia Y. Zhu C. (2025). Effect of electroacupuncture on E/I balance of prefrontal cortex and hippocampal pyramidal neurons in anxiety-like mice. Zhen Ci Yan Jiu 50, 1055–1063. doi: 10.13702/j.1000-0607.20240586 40962729

[ref35] Maeda Y. Kettner N. Lee J. Kim J. Cina S. Malatesta C. . (2013). Acupuncture-evoked response in somatosensory and prefrontal cortices predicts immediate pain reduction in carpal tunnel syndrome. Evidence-Based Complement. Alter. Med. 2013:795906. doi: 10.1155/2013/795906, 23843881 PMC3703406

[ref36] Maeda Y. Kim H. Kettner N. Kim J. Cina S. Malatesta C. . (2017). Rewiring the primary somatosensory cortex in carpal tunnel syndrome with acupuncture. Brain 140, 914–927. doi: 10.1093/brain/awx015, 28334999 PMC5837382

[ref37] Napadow V. Makris N. Liu J. Kettner N. W. Kwong K. K. Hui K. K. S. (2005). Effects of electroacupuncture versus manual acupuncture on the human brain as measured by fmri. Hum. Brain Mapp. 24, 193–205. doi: 10.1002/hbm.20081, 15499576 PMC6871725

[ref38] Ouyang G. Jia S. Wang F. Shi Y. Gao Z. (2005). Effects of electroacupuncture of different frequencies on cerebral blood perfusion and cerebral function in the patient of stroke. Zhongguo Zhen Jiu 25, 776–778. Chinese 16335203

[ref39] Qi D. Wu S. Zhang Y. Li W. (2016). Electroacupuncture analgesia with different frequencies is mediated via different opioid pathways in acute visceral hyperalgesia rats. Life Sci. 160, 64–71. doi: 10.1016/j.lfs.2016.06.025, 27370939

[ref40] Qiu Z. Ding Y. Cui L. Hu M. Ding M. (2015). The expression patterns of c-fos and c-Jun induced by different frequencies of electroacupuncture in the brain. Evid. Based Complement. Alternat. Med. 2015:343682. doi: 10.1155/2015/343682, 26491460 PMC4603316

[ref41] Rodrigues J. M. Ventura C. Abreu M. Santos C. Monte J. Machado J. P. . (2023). Electro-acupuncture effects measured by functional magnetic resonance imaging-a systematic review of randomized clinical trials. Healthcare (Basel, Switzerland) 12:2. doi: 10.3390/healthcare12010002, 38200908 PMC10778902

[ref42] Shen Z. Chang W. Liang L. Zhang J. Wang Y. Yin X. . (2025). Electroacupuncture effects on trigeminal neuralgia with comorbid anxiety and depression: the role of frequency and acupoint specificity. FASEB J. 39:e70337. doi: 10.1096/fj.202402461RR, 39840659

[ref43] Shen E. Lai Y. (2007). The efficacy of frequency-specific acupuncture stimulation on extracellular dopamine concentration in striatum--a rat model study. Neurosci. Lett. 415, 179–184. doi: 10.1016/j.neulet.2007.01.022, 17280787

[ref44] Shukla S. Torossian A. Duann J. Leung A. (2011). The analgesic effect of electroacupuncture on acute thermal pain perception--a central neural correlate study with fmri. Mol. Pain 7:45. doi: 10.1186/1744-8069-7-45, 21645415 PMC3130679

[ref45] Wang Y. Li W. Zhang K. Wang H. Kong L. (2016). Effect of electroacupuncture with different frequencies on learning and memory ability and synapse in hippocamp in rats with Alzheimer’s disease. Zhongguo Kang Fu Li Lun Yu Shi Jian. 22, 635–639. doi: 10.3969/j.issn.1006-9771.2016.06.003

[ref46] Wang X. Luo P. Zhang L. Sun J. Cao J. Lei Z. . (2024). Altered functional brain activity in first-episode major depressive disorder treated with electro-acupuncture: a resting-state functional magnetic resonance imaging study. Heliyon 10:e29613. doi: 10.1016/j.heliyon.2024.e29613, 38681626 PMC11053281

[ref47] Wang Y. Shen Y. Lin H. Li Z. Chen Y. Wang S. (2016). Large-conductance ca(2+)-activated k(+) channel involvement in suppression of cerebral ischemia/reperfusion injury after electroacupuncture at shuigou (gv26) acupoint in rats. Neural Regen. Res. 11, 957–962. doi: 10.4103/1673-5374.184495, 27482225 PMC4962594

[ref48] Wang Y. Wang X. Han J. (1998). Effects of 2 hz and 100 hz electroacupuncture tolerance on the transcription of central nervous system κ-opioid receptor mrna. Beijing Yi Ke Da Xue Xue Bao. 30, 1–04.

[ref49] Wang G. Wu L. Yu P. Li Y. Ping X. Cui C. (2011). Multiple 100 hz electroacupuncture treatments produced cumulative effect on the suppression of morphine withdrawal syndrome: central preprodynorphin mrna and p-creb implicated. Peptides 32, 713–721. doi: 10.1016/j.peptides.2010.12.006, 21167242

[ref50] Wei J. Hu X. Zhang B. Liu L. Chen K. So K. . (2021). Electroacupuncture activates inhibitory neural circuits in the somatosensory cortex to relieve neuropathic pain. iScience 24:102066. doi: 10.1016/j.isci.2021.102066, 33554069 PMC7859294

[ref51] Xiang R. Liang F. Liu S. Xie M. Wu F. Zhang S. . (2026). Effect of different frequencies electroacupuncture at "zusanli"(st36) on visceral sensitivity and adrenocorticotropin releasing factor expression in IBS mice. Zhen Ci Yan Jiu 51, 323–331. doi: 10.13702/j.1000-0607.20250106 41839582

[ref52] Xiao Y. Wu X. Deng X. Huang L. Zhou Y. Yang X. (2013). Optimal electroacupuncture frequency for maintaining astrocyte structural integrity in cerebral ischemia. Neural Regen. Res. 8, 1122–1131. doi: 10.3969/j.issn.1673-5374.2013.12.007, 25206406 PMC4145895

[ref53] Xiong F. Li S. Q. Wang Y. Wu X. Mu K. T. Zheng C. H. . (2020). Comparision of brain effects with different intensities of electroacupuncture at shangjuxu (ST37) shown by fMRI. Shi Zhen Guo Yi Guo Yao. 31, 749–752. doi: 10.3969/j.issn.1008-0805.2020.03.082

[ref54] Xiong F. Li S. Q. Wu X. Mu K. T. Liu Y. Zheng C. H. . (2019). Functional magnetic resonance imaging comparison of the post-stimulation effects of electroacupuncture at Shangjuxu (ST37) with different intensities. Zhongguo Zhong Yi Yao Xin Xi Za Zhi. 26, 26–30. doi: 10.3969/j.issn.1005-5304.2019.12.007

[ref55] Xu M. Lin R. Wen H. Wang Y. Wong J. Peng Z. . (2024). Electroacupuncture enhances the functional connectivity of limbic system to neocortex in the 5xfad mouse model of alzheimer's disease. Neuroscience 544, 28–38. doi: 10.1016/j.neuroscience.2024.02.025, 38423162

[ref56] Yao S. Liu Y. Cui S. Li H. Ji C. Yuan S. . (2020). Effect of different frequencies of electroacupuncture on post-stroke dysphagia in mice. J. Mol. Neurosci. 70, 1871–1879. doi: 10.1007/s12031-020-01580-1, 32519209

[ref57] Yu Y. Cao X. Dong C. Hou X. Zhang T. Zhang X. . (2026). Effects of electroacupuncture with different waveforms on nlrp3 inflammasome and pyroptosis in the cerebral cortex of rats with cerebral ischemia-reperfusion injury. Zhongguo Zhen Jiu 46, 393–401. doi: 10.13703/j.0255-2930.20241207-k0004 41839600

[ref58] Yu L. Li L. Qin Q. Yu Y. Cui X. Rong P. . (2018). Electroacupuncture inhibits visceral nociception via somatovisceral interaction at subnucleus reticularis dorsalis neurons in the rat medulla. Front. Neurosci. 12:775. doi: 10.3389/fnins.2018.00775, 30425615 PMC6218567

[ref59] Zha B. Zhang Y. Shi F. Cheng L. Rong Z. Yu L. . (2024). Modulations of resting-static functional connectivity on insular by electroacupuncture in subjective tinnitus. Front. Neurol. 15:1373390. doi: 10.3389/fneur.2024.1373390, 38585348 PMC10995322

[ref60] Zhang Y. Chen A. Tang C. Zhang Y. Yin H. Chen S. (2013). Clinical observation on electroacupuncture for arousing consciousness of comatose patients with severe trauma brain injury. Zhen Ci Yan Jiu 38, 158–162. doi: 10.13702/j.1000-0607.2013.02.017 23819221

[ref61] Zhang X. Cui H. Zheng S. Lu Y. Yuan H. Zhang L. . (2023). Electroacupuncture regulates microglial polarization via inhibiting nf-κb/cox2 pathway following traumatic brain injury. Brain Res. 1818:148516. doi: 10.1016/j.brainres.2023.148516, 37562566

[ref62] Zhang S. Gong P. Zhang J. Mao X. Zhao Y. Wang H. . (2020). Specific frequency electroacupuncture stimulation transiently enhances the permeability of the blood-brain barrier and induces tight junction changes. Front. Neurosci. 14:582324. doi: 10.3389/fnins.2020.582324, 33122995 PMC7573286

[ref63] Zhang J. Hui R. Xu J. Zhang L. Xie B. Ma C. . (2025). Low-frequency electroacupuncture attenuates methamphetamine-induced depressive-like behaviors and cognitive impairment via modulating neuroinflammation. Front. Neurol. 16:1652065. doi: 10.3389/fneur.2025.1652065, 41189656 PMC12580150

[ref64] Zhang W. Jin Z. Cui G. Zhang K. Zhang L. Zeng Y. . (2003). Relations between brain network activation and analgesic effect induced by low vs. high frequency electrical acupoint stimulation in different subjects: a functional magnetic resonance imaging study. Brain Res. 982, 168–178. doi: 10.1016/s0006-8993(03)02983-4 12915252

[ref65] Zhang R. Lao L. Ren K. Berman B. M. (2014). Mechanisms of acupuncture-electroacupuncture on persistent pain. Anesthesiology 120, 482–503. doi: 10.1097/ALN.0000000000000101, 24322588 PMC3947586

[ref66] Zhang J. Lin X. Zhou H. Chen Y. Xiao S. Jiao J. . (2018). Electroacupuncture: a new approach to open the blood-brain barrier in rats recovering from middle cerebral artery occlusion. Acupun. Med. 36, 377–385. doi: 10.1136/acupmed-2017-011496, 29903719 PMC6287560

[ref67] Zhang C. Xie M. Shen Z. Hu Y. Liu Q. Zhu X. . (2026). Electroacupuncture alleviates neuropathic pain and long-term cognitive impairment via the racc-vlpag neural circuit. J. Psychiatr. Res. 193, 267–277. doi: 10.1016/j.jpsychires.2025.11.037, 41344227

[ref68] Zhang T. Yang L. Hu R. Qiao X. Yang X. Liu X. (2007). Effect of electroacupuncture on the contents of excitatory amino acids in cerebral tissue at different time courses in rats with cerebral ischemia and reperfusion injury. Zhen Ci Yan Jiu 32, 234–236. 17907384

[ref69] Zhang Y. Zha B. Shi H. Fan Y. Zheng Y. Rong Z. . (2024). Exploring the regulatory effect of acupuncture for ningshen tongqiao on the functional connectivity between the anterior cingulate cortex and the whole brain in the patients with subjective tinnitus based on fmri. Zhongguo Zhen Jiu 44, 12–18. doi: 10.13703/j.0255-2930.20230628-0003 38191153

[ref70] Zhang J. Zhang S. Zhang H. (2009). Effect of electroacupuncture on thalamic neuronal response to visceral nociception. Euro. J. Pain (London, England) 13, 366–372. doi: 10.1016/j.ejpain.2008.04.016, 18547846

[ref71] Zhao Z. (2008). Neural mechanism underlying acupuncture analgesia. Prog. Neurobiol. 85, 355–375. doi: 10.1016/j.pneurobio.2008.05.004, 18582529

[ref72] Zhou F. Guo J. Cheng J. Wu G. Sun J. Xia Y. (2013a). Electroacupuncture and brain protection against cerebral ischemia: specific effects of acupoints. Evidence-Based Complement. Alter. Med. 2013:804397. doi: 10.1155/2013/804397, 23737846 PMC3666307

[ref73] Zhou F. Guo J. Cheng J. Wu G. Xia Y. (2011). Electroacupuncture increased cerebral blood flow and reduced ischemic brain injury: dependence on stimulation intensity and frequency. J. Appl. Physiol. 111, 1877–1887. doi: 10.1152/japplphysiol.00313.2011, 21836043 PMC3233896

[ref74] Zhou F. Guo J. Cheng J. Wu G. Xia Y. (2013b). Effect of electroacupuncture on rat ischemic brain injury: importance of stimulation duration. Evidence-Based Complement. Alter. Med. 2013:878521. doi: 10.1155/2013/878521, 23737851 PMC3666426

[ref75] Zhou M. Zhang Q. Huo M. Song H. Chang H. Cao J. . (2023). The mechanistic basis for the effects of electroacupuncture on neuropathic pain within the central nervous system. Biomed. Pharmacother. 161:114516. doi: 10.1016/j.biopha.2023.114516, 36921535

