# Supplementary material for: How electroacupuncture parameters shape brain responses
Source: Front Neurosci. 2026 Oct 8;20:1959062. doi: 10.3389/fnins.2026.1959062 (PMC13687710; doi:10.3389/fnins.2026.1959062)
Supplement: Supplementary file 1 [file Data_Sheet_1.DOCX]

**Supplementary Material**

# 1. Review design and scope

This article is a narrative review supported by a structured literature search. The search was used to identify and organize a heterogeneous body of human and animal evidence; it was not used to support a meta-analysis, a pooled effect estimate, or a formal cross-design risk-of-bias score. Needle-based electroacupuncture (EA) was the primary intervention. Transcutaneous electrical acupoint stimulation (TEAS) was retained only as complementary evidence and was coded separately.

# 2. Information sources and database-specific search strategies

PubMed/MEDLINE, Embase, Web of Science Core Collection, China National Knowledge Infrastructure (CNKI), and SinoMed were searched from database inception through 4 August 2026. Primary searches combined intervention, electrical-parameter, and brain-response concepts. Targeted supplementary searching and reference-list screening were used to identify acupoint/site studies and mechanistically informative records that were not captured by the broad parameter query.

## Supplementary Table S1. Search sources and final exported records

| **Source** | **Coverage** | **Search field/interface** | **Records** |
| --- | --- | --- | --- |
| PubMed/MEDLINE | Inception–4 Aug 2026 | MeSH and title/abstract fields; main plus targeted query | 595 |
| Embase | Inception–4 Aug 2026 | Title/abstract/keyword | 156 |
| Web of Science Core Collection | Inception–4 Aug 2026 | Topic | 234 |
| CNKI | Inception–4 Aug 2026 | Subject | 152 |
| SinoMed | Inception–4 Aug 2026 | Advanced Search; core/subject field | 81 |
| Total before cross-database deduplication | — | — | 1,218 |

*Note. The PubMed/MEDLINE total is the unique export after the main and targeted PubMed searches were merged and deduplicated within the citation manager. Search-result counts may change slightly as database indexing is updated; the reported values are the saved counts used for the review.*

## 2.1 PubMed/MEDLINE: main search

Fields: MeSH and title/abstract. Date limit included in the search string.

(("Electroacupuncture"[Mesh] OR electroacupuncture[tiab] OR electro-acupuncture[tiab] OR "electrical acupoint stimulation"[tiab] OR "electrical acupuncture"[tiab] OR "transcutaneous electrical acupoint stimulation"[tiab] OR "transcutaneous electric acupoint stimulation"[tiab]) AND (frequency[tiab] OR frequencies[tiab] OR intensity[tiab] OR intensities[tiab] OR amplitude[tiab] OR waveform*[tiab] OR "pulse width"[tiab] OR "pulse duration"[tiab] OR "stimulation duration"[tiab] OR "treatment duration"[tiab]) AND (brain[tiab] OR cerebral[tiab] OR cortex[tiab] OR cortical[tiab] OR hippocamp*[tiab] OR hypothalam*[tiab] OR thalam*[tiab] OR brainstem[tiab] OR "functional magnetic resonance"[tiab] OR fMRI[tiab] OR EEG[tiab] OR electroencephalog*[tiab] OR "neural circuit"[tiab] OR "blood-brain barrier"[tiab])) AND ("1900/01/01"[Date - Publication] : "2026/08/04"[Date - Publication])

## 2.2 PubMed/MEDLINE: targeted supplementary search

Purpose: retrieval of single-protocol mechanisms, acupoint/site specificity, and named brain-region/circuit studies not captured by the main query.

(("Electroacupuncture"[Mesh] OR electroacupuncture[tiab] OR electro-acupuncture[tiab] OR "electrical acupoint stimulation"[tiab] OR "electrical acupuncture"[tiab] OR "transcutaneous electrical acupoint stimulation"[tiab] OR "transcutaneous electric acupoint stimulation"[tiab]) AND ("central serotonergic system"[tiab] OR "conditioned place preference"[tiab] OR "morphine withdrawal syndrome"[tiab] OR ("brain protection"[tiab] AND "cerebral ischemia"[tiab]) OR "acute visceral hyperalgesia"[tiab] OR ("regional homogeneity"[tiab] AND fMRI[tiab]) OR ("large-conductance"[tiab] AND "cerebral ischemia"[tiab]) OR ("point selection"[tiab] AND "stimulation frequency"[tiab]) OR ("brain connectivity"[tiab] AND (Crohn*[tiab] OR "carpal tunnel"[tiab])) OR "swallowing cortex"[tiab] OR "subnucleus reticularis dorsalis"[tiab] OR "excitatory and inhibitory neurotransmitters"[tiab] OR "point specificity"[tiab] OR "npy-expressing sympathetic pathways"[tiab] OR "inhibitory neural circuits"[tiab] OR "vagal-adrenal axis"[tiab] OR "s1 brain connectivity"[tiab] OR "infralimbic medial prefrontal cortex"[tiab] OR ("limbic system"[tiab] AND neocortex[tiab]) OR ("functional connectivity"[tiab] AND insular[tiab]) OR ("visceral pain"[tiab] AND intensit*[tiab]) OR "racc-vlpag"[tiab])) AND ("1900/01/01"[Date - Publication] : "2026/08/04"[Date - Publication])

## 2.3 Web of Science Core Collection

Field: Topic. Timespan: database inception to 4 August 2026.

TS=(electroacupuncture OR "electro-acupuncture" OR "electrical acupoint stimulation" OR "transcutaneous electrical acupoint stimulation") AND TS=(frequency OR "current intensity" OR amplitude OR waveform OR "pulse width" OR "pulse duration" OR "stimulation duration" OR "treatment duration") AND TS=("functional magnetic resonance imaging" OR electroencephalography OR "neuronal activity" OR "neural oscillation" OR "functional connectivity" OR neurotransmitter OR "c-Fos" OR "c-Jun" OR "cerebral blood flow" OR "blood-brain barrier" OR neuroinflammation OR neuroplasticity OR "neural circuit")

## 2.4 Embase

Fields: title, abstract, and author keywords. Date limited through 4 August 2026 in the platform.

('electroacupuncture':ti,ab,kw OR 'electro-acupuncture':ti,ab,kw OR 'electrical acupoint stimulation':ti,ab,kw OR 'transcutaneous electrical acupoint stimulation':ti,ab,kw) AND ('frequency':ti,ab,kw OR 'current intensity':ti,ab,kw OR 'amplitude':ti,ab,kw OR 'waveform':ti,ab,kw OR 'pulse width':ti,ab,kw OR 'pulse duration':ti,ab,kw OR 'stimulation duration':ti,ab,kw OR 'treatment duration':ti,ab,kw) AND ('functional magnetic resonance imaging':ti,ab,kw OR 'electroencephalography':ti,ab,kw OR 'neuronal activity':ti,ab,kw OR 'neural oscillation':ti,ab,kw OR 'functional connectivity':ti,ab,kw OR 'neurotransmitter':ti,ab,kw OR 'c-Fos':ti,ab,kw OR 'c-Jun':ti,ab,kw OR 'cerebral blood flow':ti,ab,kw OR 'blood-brain barrier':ti,ab,kw OR 'neuroinflammation':ti,ab,kw OR 'neuroplasticity':ti,ab,kw OR 'neural circuit':ti,ab,kw)

## 2.5 CNKI

Field: subject. Date limited through 4 August 2026 in the platform.

SU%=(电针+电针疗法+电穴位刺激+穴位电刺激+经皮穴位电刺激) AND SU%=(频率+电流强度+振幅+波形+脉宽+脉冲宽度+脉冲持续时间+刺激持续时间+治疗持续时间) AND SU%=(功能磁共振成像+脑电图+神经元活动+神经振荡+功能连接+神经递质+'c-Fos'+'c-Jun'+脑血流+血脑屏障+神经炎症+神经可塑性+神经环路)

## 2.6 SinoMed

Advanced Search. Date limited through 4 August 2026 in the platform.

Advanced Search; the following three rows were entered in the same core/subject field and connected with AND.
Row 1: 电针 OR 电针疗法 OR 电穴位刺激 OR 穴位电刺激 OR 经皮穴位电刺激
Row 2: 频率 OR 电流强度 OR 振幅 OR 波形 OR 脉宽 OR 脉冲宽度 OR 脉冲持续时间 OR 刺激持续时间 OR 治疗持续时间
Row 3: 功能磁共振成像 OR 脑电图 OR 神经元活动 OR 神经振荡 OR 功能连接 OR 神经递质 OR c-Fos OR c-Jun OR 脑血流 OR 血脑屏障 OR 神经炎症 OR 神经可塑性 OR 神经环路

# 3. Eligibility criteria

## Supplementary Table S2. Eligibility criteria for the evidence synthesis

| **Domain** | **Included** | **Excluded or handled separately** |
| --- | --- | --- |
| Publication type | Original human or animal research | Reviews and methodological articles were used only for context |
| Intervention | Clearly defined needle-based EA protocol | Manual acupuncture alone; inadequately described electrical protocol |
| Complementary intervention | TEAS only when parameters were directly compared and cerebral outcomes were assessed | TEAS was not pooled with needle-based EA |
| Outcome | At least one brain-level outcome: regional activity/connectivity, neuronal or oscillatory activity, neurochemical signaling, neurovascular/neuroimmune response, plasticity, or a defined brain region/circuit | Peripheral or spinal outcomes only; clinical or behavioral outcomes without a corresponding brain measure |
| Direct evidence | Within-study comparison of at least two levels or choices in one focal dimension for the same brain outcome | Between-study contrasts were not treated as direct parameter evidence |
| Single-protocol evidence | Clearly defined protocol with direct brain measurement/manipulation and non-redundant anatomical or mechanistic information | Not used to infer parameter specificity or superiority; illustrative rather than exhaustive |

# 4. Record management, screening, and data extraction

Search records were imported into NoteExpress (version 4.3.0.10710). Duplicate records were identified using PMID, DOI, and title information and were then checked manually when metadata were incomplete or inconsistent. Title-and-abstract screening was conducted by CT and FL. Full-text assessment and data extraction were conducted by CT and QL. CT verified the final extracted dataset. Discrepancies were resolved through discussion among the three authors; no unresolved disagreements remained.

The extraction form captured citation and publication details; human population or animal species/model; sample size as reported; study design and comparator/control; randomization and blinding when reported; EA or TEAS mode; stimulation site/acupoint and laterality; frequency; current amplitude and relation to sensory or motor threshold; pulse width; waveform, train structure, duty cycle, and switching interval; session duration, needle depth, and number of sessions; timing of outcome measurement; brain-assessment or manipulation method; brain region, network, cell population, receptor, or circuit; key quantitative or directional result; correction for multiple comparisons when relevant; and important interpretive limitations. Fields not reported by the primary article were coded as not reported and were not inferred.

## Study-selection counts

1. Records identified across five databases: 1,218.
2. Duplicate records removed: 271 records, representing 168 duplicate clusters.
3. Records screened by title and abstract: 947; excluded at this stage: 384.
4. Full-text reports assessed: 563; excluded after full-text assessment: 488.
5. Sources retained in the narrative review: 75; core brain-level evidence: 69; contextual sources: 6.

# 5. Evidence classification and coding rules

## Supplementary Table S3. Evidence categories and permissible inferences

| **Category** | **Operational rule** | **Permissible inference** | **Not permissible** |
| --- | --- | --- | --- |
| Direct comparative | Within-study comparison of two or more levels/choices in a focal intervention dimension for the same brain outcome | Parameter- or site-related difference under the tested conditions | Universal superiority; evidence strength based only on significance |
| Composite-protocol contrast | A direct comparison in which another major electrical or anatomical feature also changed | Difference between the tested composite protocols | Attribution to one isolated parameter |
| Single-protocol needle EA | One defined needle-based EA protocol without a within-study comparison of the focal dimension | Association or causal involvement of a region, network, receptor, cell population, or circuit under that protocol | Parameter specificity or superiority |
| Complementary TEAS | Transcutaneous acupoint stimulation with a direct parameter comparison and cerebral outcome | Cerebral response to TEAS under the tested protocols | Direct equivalence to needle-based EA |
| Contextual source | Review, methodological paper, or clinically relevant report used only for interpretation | Background or translational context | Counted as core mechanistic evidence |

# 6. Reconciliation of study and category counts

The unit used for evidence coding was the study–outcome–comparison, whereas the total included evidence base was counted by unique report. Panel A of Figure 2 contains 53 unique needle-EA reports but 56 focal-dimension assignments. The difference arises because Fang et al. (69) contributed to both frequency and acupoint/site evidence, and Liu et al. (9) and Liu et al. (70) each contributed to both current-intensity and acupoint/site evidence. Panel B contains 16 single-protocol needle-EA entries and three TEAS comparisons (19 entries). Three reports—references 18, 49, and 70—contributed to both direct-comparative and single-protocol coding because different experiments or outcomes within the same report supported different evidence roles. Therefore, the unique core evidence total is 53 + 16 + 3 − 3 = 69, not 72.

*Context-only references were 1, 2, 10, 11, 54, and 75. They were not counted in the 69-report core evidence synthesis.*

# 7. Interpretation of direction, magnitude, and evidence strength

Symbols such as ↑, ↓, >, and NS record the direction or reported statistical status of an outcome only. They do not represent effect size, study quality, causal certainty, or evidential weight. Study-level interpretation considered the experimental design, sample size, comparator/control, measurement modality, multiple testing, randomization or blinding when reported, and whether the study demonstrated association or causal involvement. Because designs and endpoints were highly heterogeneous, no numerical evidence score or pooled ranking was assigned. Exact quantitative results were retained in the narrative when they were central and comparable; otherwise, the primary article remains the source for assay-specific numerical estimates.

# Supplementary Table S4. Composite-protocol contrasts and co-varying characteristics

| **Study** | **Focal dimension** | **Protocols compared** | **Co-varying characteristic(s)** | **Interpretive use** |
| --- | --- | --- | --- | --- |
| Chai et al., 2025 (6) | Frequency | 2/15 Hz vs 50 Hz; manual-acupuncture comparator | Train pattern differed (alternating 2/15 Hz vs single-frequency 50 Hz); contrasts with the manual-acupuncture group also changed intervention modality and electrical exposure (needle stimulation without electrical current). Bilateral ST36 + PC6 and propofol anesthesia were common. | Composite protocol; not an isolated frequency contrast for every outcome |
| Fang et al., 2017 (69) | Frequency; acupoint/site | ST36 vs ST25 across 2, 100, and 2/100 Hz | Acupoint and frequency were crossed: hindlimb ST36 versus abdominal ST25 at 2, 100, or alternating 2/100 Hz (switched every 3 s). The 2/100-Hz condition also changed switching pattern/train structure; intensity (1–2 mA), pulse width (0.5 ms), and 30-min duration were common. | Supports a point–frequency interaction, not a site-independent frequency effect |
| Liu et al., 2020 (9) | Current intensity; acupoint/site | Low-intensity ST36 vs high-intensity ST25 | Current amplitude and anatomical site changed together: low-intensity hindlimb ST36 (0.5 mA) versus higher-intensity abdominal ST25 (1–3 mA). Local tissue and accessible afferents therefore also differed; frequency (10 Hz), pulse width (50 μs), and duration (15 min) were common. | Pathway difference attributed to the composite site–intensity protocol |
| Liu et al., 2021 (70) | Current intensity; acupoint/site | 0.5-mA ST36 vs 3.0-mA ST36/ST25 at 10 Hz | Amplitude, site, and stimulated tissue compartment varied: 0.5-mA deep ST36 stimulation recruited PROKR2-positive deep-fascial afferents, whereas 3.0-mA stimulation at ST36 or abdominal ST25 recruited broader PROKR2-independent spinal-sympathetic pathways. All used 10 Hz, 50-μs pulses for 15 min. | Separates afferent dependence at ST36; cross-site contrasts remain composite |
| Chen et al., 2016 (59) | Waveform | Continuous 2 Hz vs intermittent 2/0 Hz vs dilatational 2/15 Hz | The waveform labels incorporated several temporal changes: continuous 2 Hz, interrupted 2-Hz trains (2/0 Hz), and alternating 2/15-Hz dilatational stimulation differed in interruption, switching, component frequency, and train structure. GV20 + ST36 and 30-min pretreatment were common. | Difference between temporal protocols; not waveform isolated from frequency |
| Yu et al., 2026 (60) | Waveform | Continuous 15 Hz vs discontinuous 15-Hz trains vs dense–disperse 3/15 Hz | All groups used GV20 + GV14 + bilateral ST36 for 20 min/day for 7 days, but temporal delivery differed: uninterrupted 15 Hz, 15-Hz trains with ≈1.5-s pauses, or alternating 3/15-Hz dense–disperse stimulation. Interruption, component frequency, and switching pattern therefore changed together. | Composite temporal-protocol comparison |
| Fu et al., 2023 (68) | Acupoint/site | HT5 + GB20 vs HT5 alone vs GB20 alone | Single-site HT5 or GB20 was compared with dual-site HT5 + GB20, so the number and anatomical distribution of stimulated sites changed together. Frequency (10 Hz), continuous wave, intensity (0.2–0.6 mA titrated to sensation), and 30-min duration were otherwise matched. | Point-combination effect; cannot isolate one point from total stimulation extent |

# Supplementary Table S5. Complementary TEAS evidence retained separately

| **Study** | **Design/population** | **TEAS comparison** | **Brain assessment** | **Main interpretive point** |
| --- | --- | --- | --- | --- |
| Zhang et al., 2003 (3) | Human; between-group comparison | 2 Hz/0.6-ms pulse width vs 100 Hz/0.2-ms pulse width | BOLD fMRI | Frequency and pulse width co-varied; cerebral recruitment differed between composite TEAS protocols |
| Jiang et al., 2014 (50) | Human | 2 vs 100 Hz | Perfusion fMRI | Both showed biphasic CBF responses; 2 Hz was broader cortically, whereas 100 Hz emphasized ventral striatum/dACC |
| Lopes Alves et al., 2025 (51) | Healthy volunteers; semi-randomized, placebo-controlled crossover | 2.5 vs 10 pulses/s | EEG source localization | Post-stimulation theta and alpha source changes differed by pulse rate |

# Abbreviations

ACC, anterior cingulate cortex; BOLD, blood oxygen level-dependent; CBF, cerebral blood flow; CNKI, China National Knowledge Infrastructure; dACC, dorsal anterior cingulate cortex; EA, electroacupuncture; EEG, electroencephalography; FC, functional connectivity; fMRI, functional magnetic resonance imaging; NR, not reported; TEAS, transcutaneous electrical acupoint stimulation.
